# Supplementary figures and images for: A delta-tubulin/epsilon-tubulin/Ted protein complex is required for centriole architecture
Source: eLife. 2025 Mar 11;13:RP98704. doi: 10.7554/eLife.98704 (PMC11896610; doi:10.7554/eLife.98704)

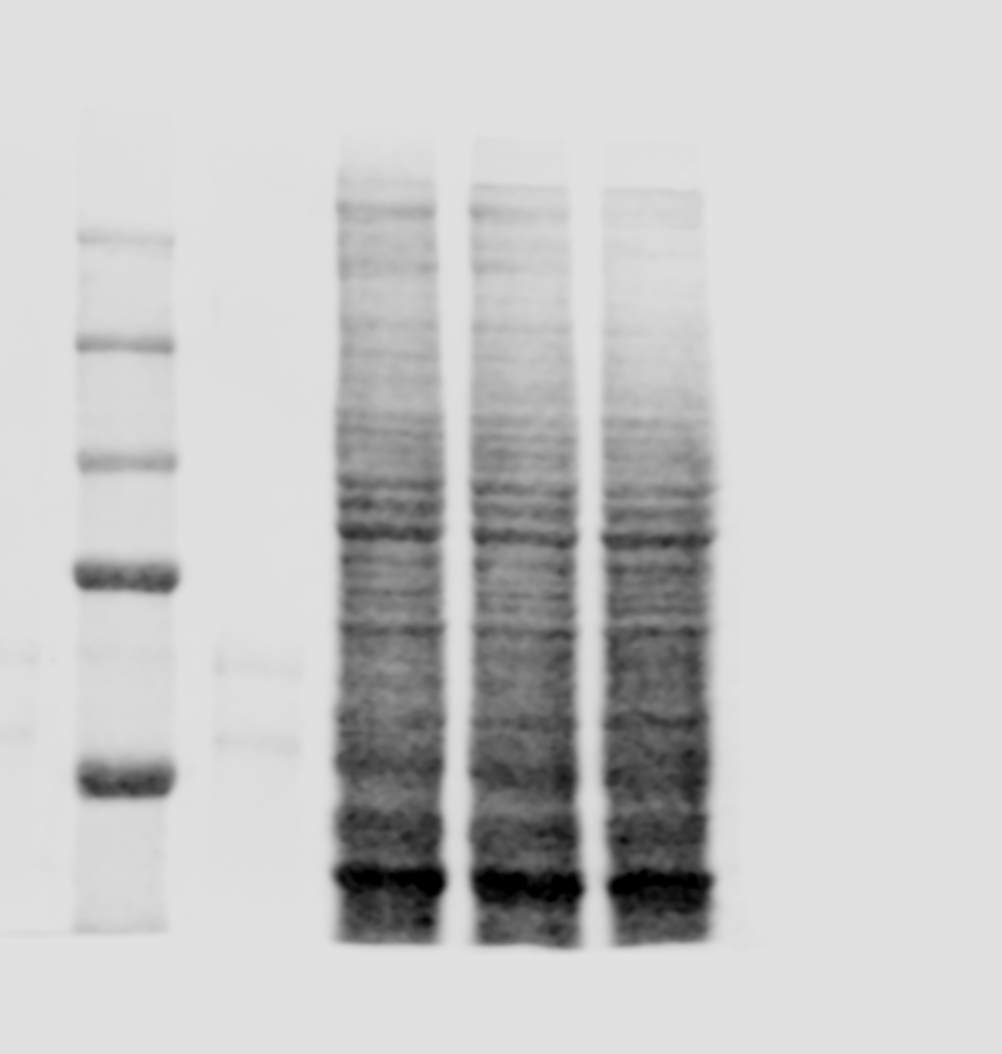

Supplement: Figure 1—figure supplement 1—source data 1. [file elife-98704-fig1-figsupp1-data1.zip › Fig 1 - Figure supplement 1 - source data 1/20240730-JWA13-Tedc1blot-Revert-unlabeled.tif]

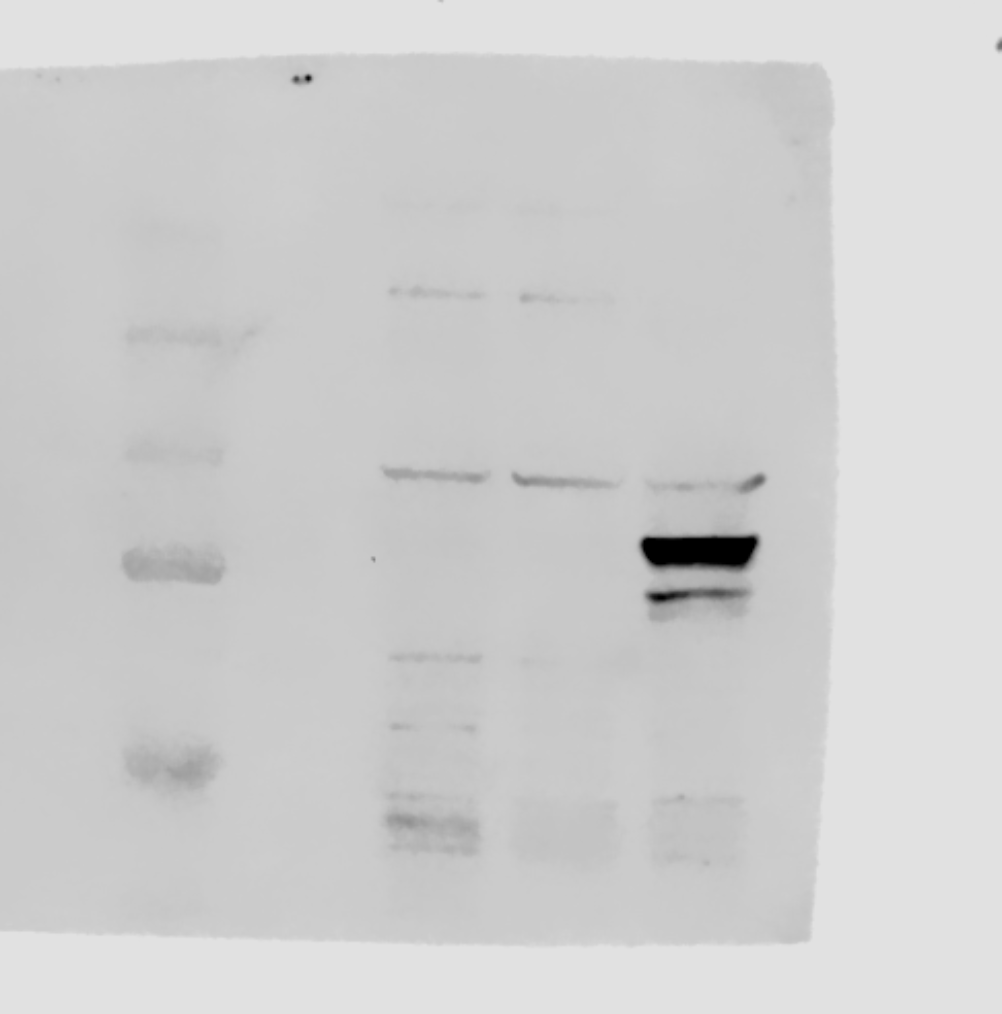

Supplement: Figure 1—figure supplement 1—source data 1. [file elife-98704-fig1-figsupp1-data1.zip › Fig 1 - Figure supplement 1 - source data 1/20240730-JWA13-Tedc1blot-no label.tif]

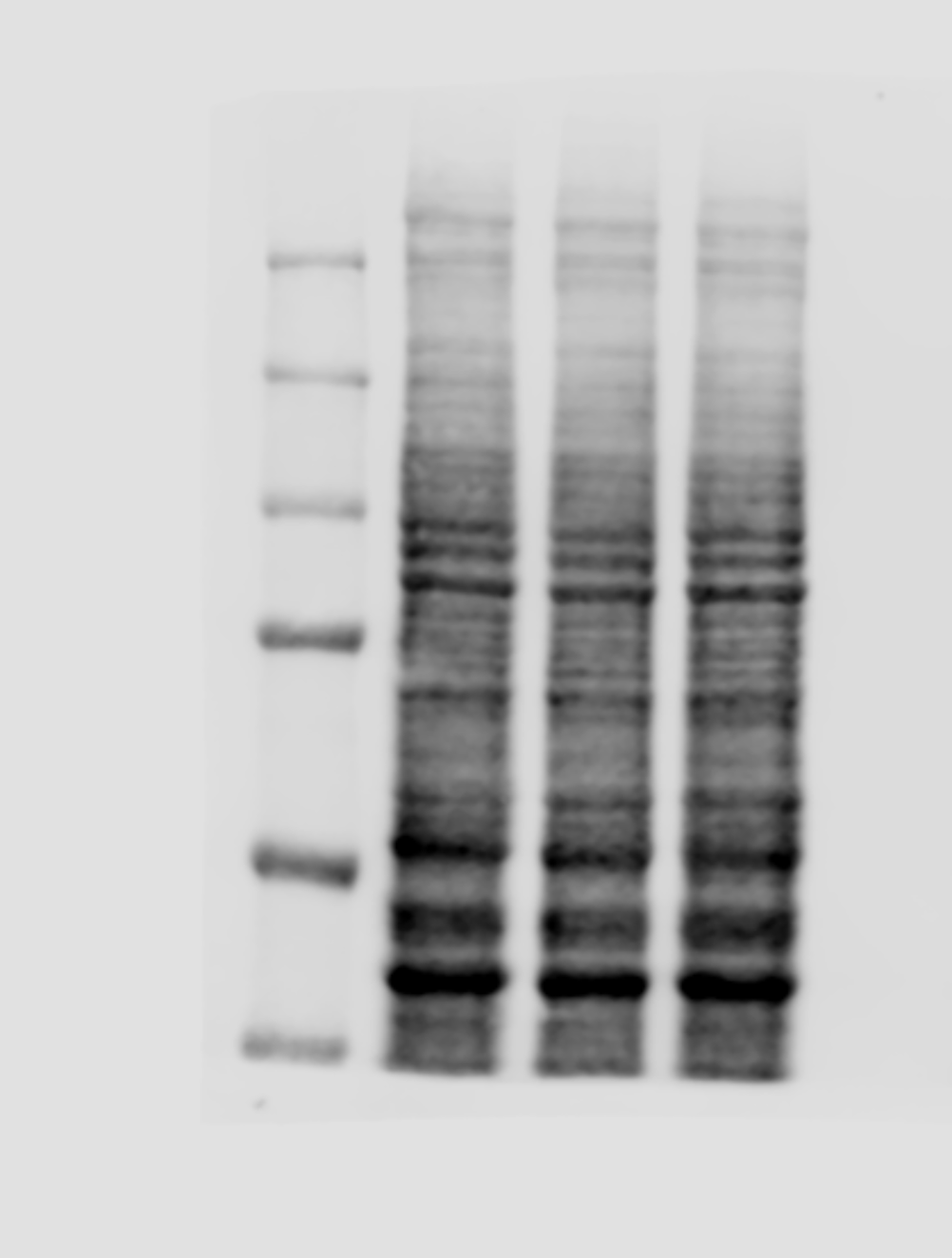

Supplement: Figure 1—figure supplement 1—source data 1. [file elife-98704-fig1-figsupp1-data1.zip › Fig 1 - Figure supplement 1 - source data 1/20240730-JWA13-Tedc2-revert-unlabeled.tif]

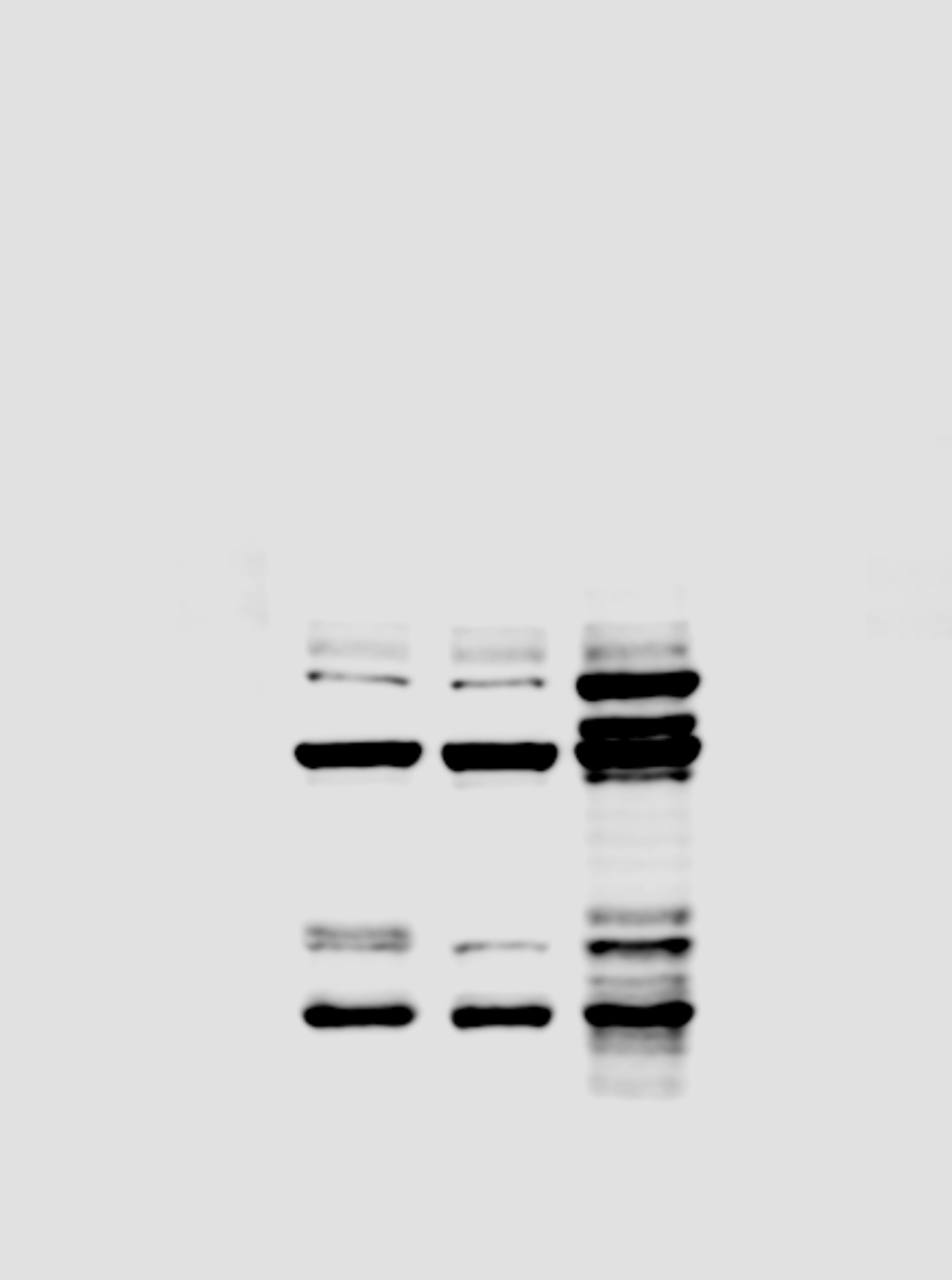

Supplement: Figure 1—figure supplement 1—source data 1. [file elife-98704-fig1-figsupp1-data1.zip › Fig 1 - Figure supplement 1 - source data 1/20240730-JWA13-Tedc2-unlabeled.tif]

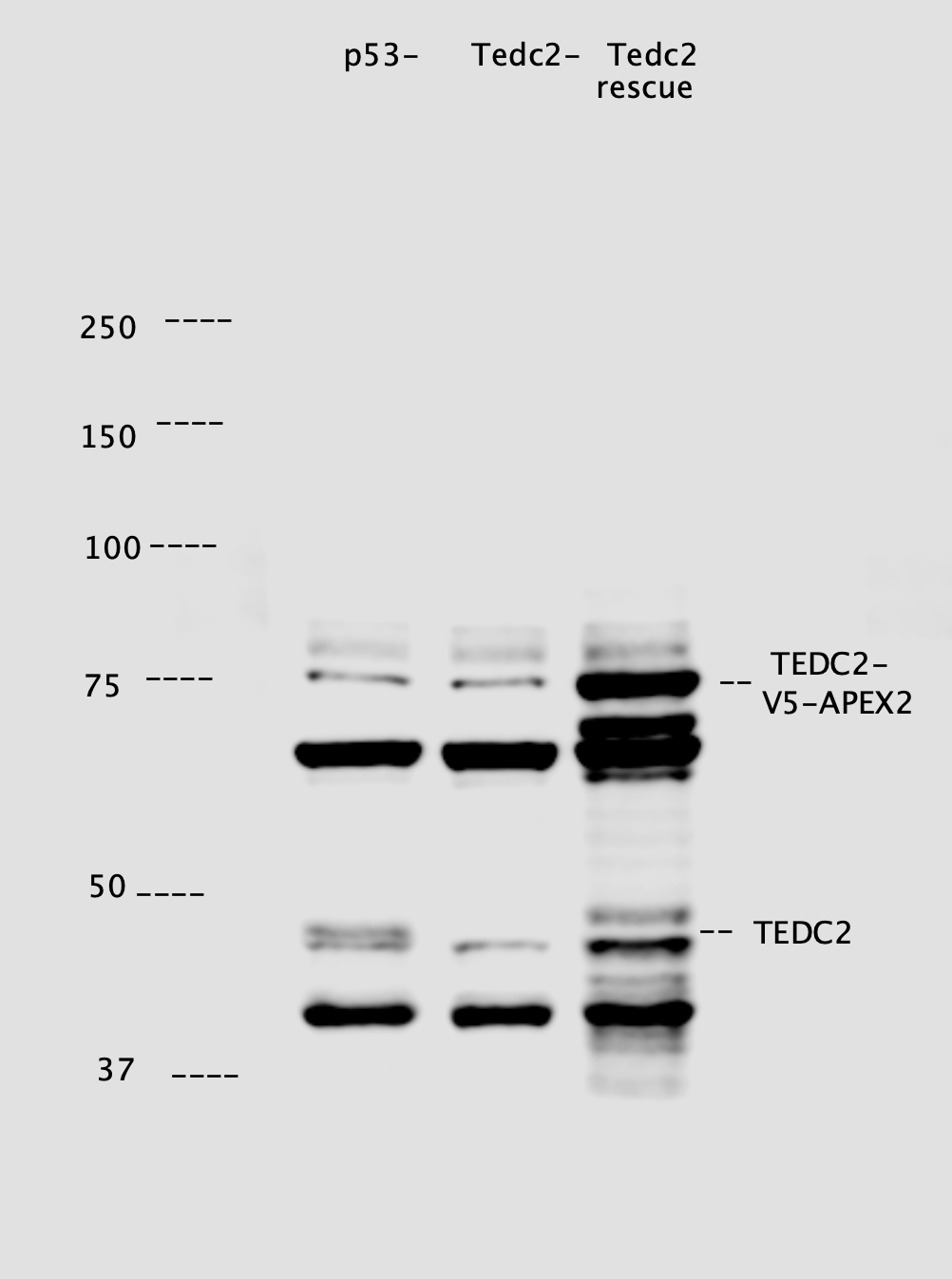

Supplement: Figure 1—figure supplement 1—source data 2. [file elife-98704-fig1-figsupp1-data2.zip › Fig 1 - Figure supplement 1 - source data 2/20240730-JWA13-Tedc2-labeled.tif]

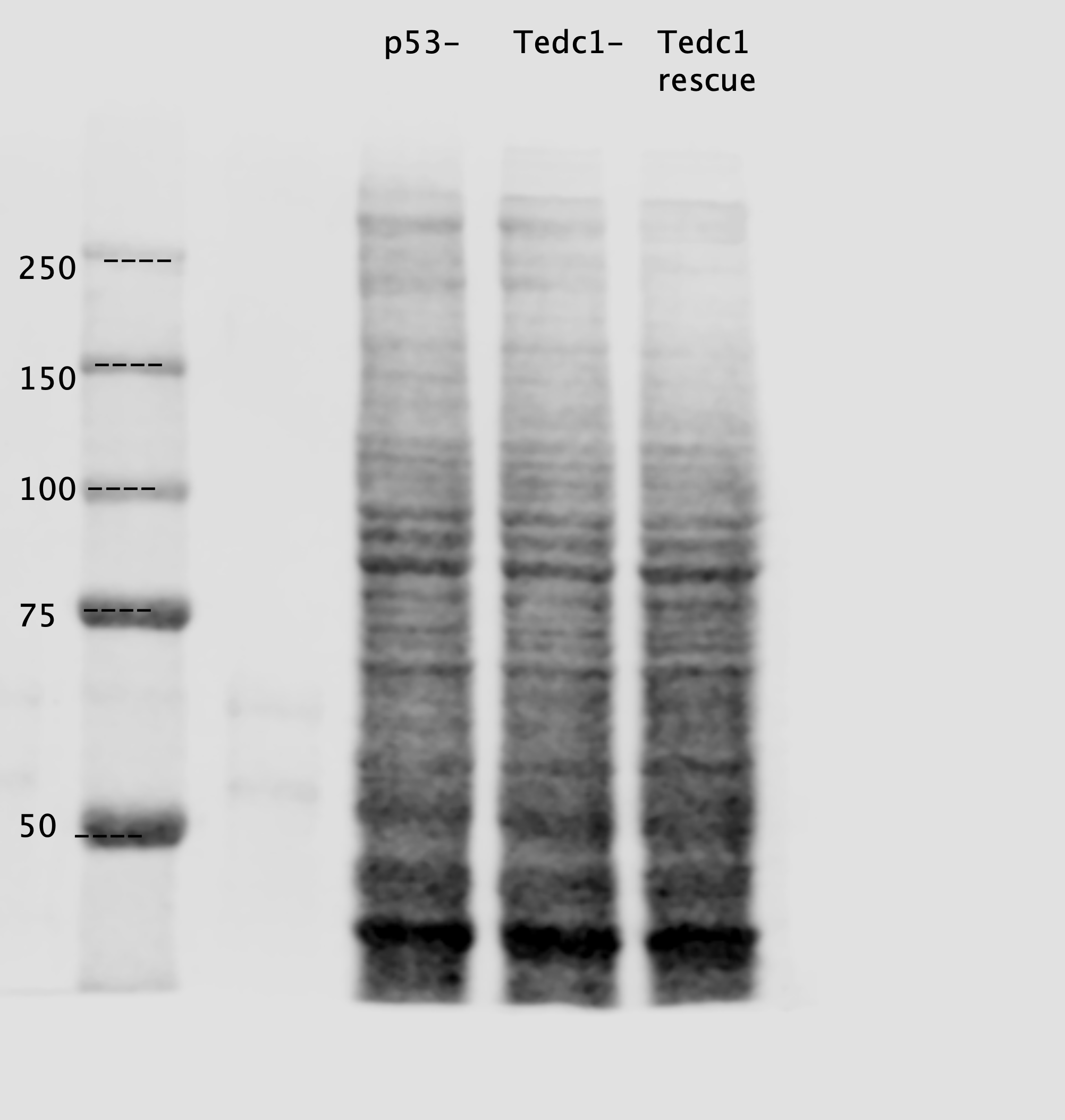

Supplement: Figure 1—figure supplement 1—source data 2. [file elife-98704-fig1-figsupp1-data2.zip › Fig 1 - Figure supplement 1 - source data 2/20240730-JWA13-Tedc1blot-Revert-labeled.tif]

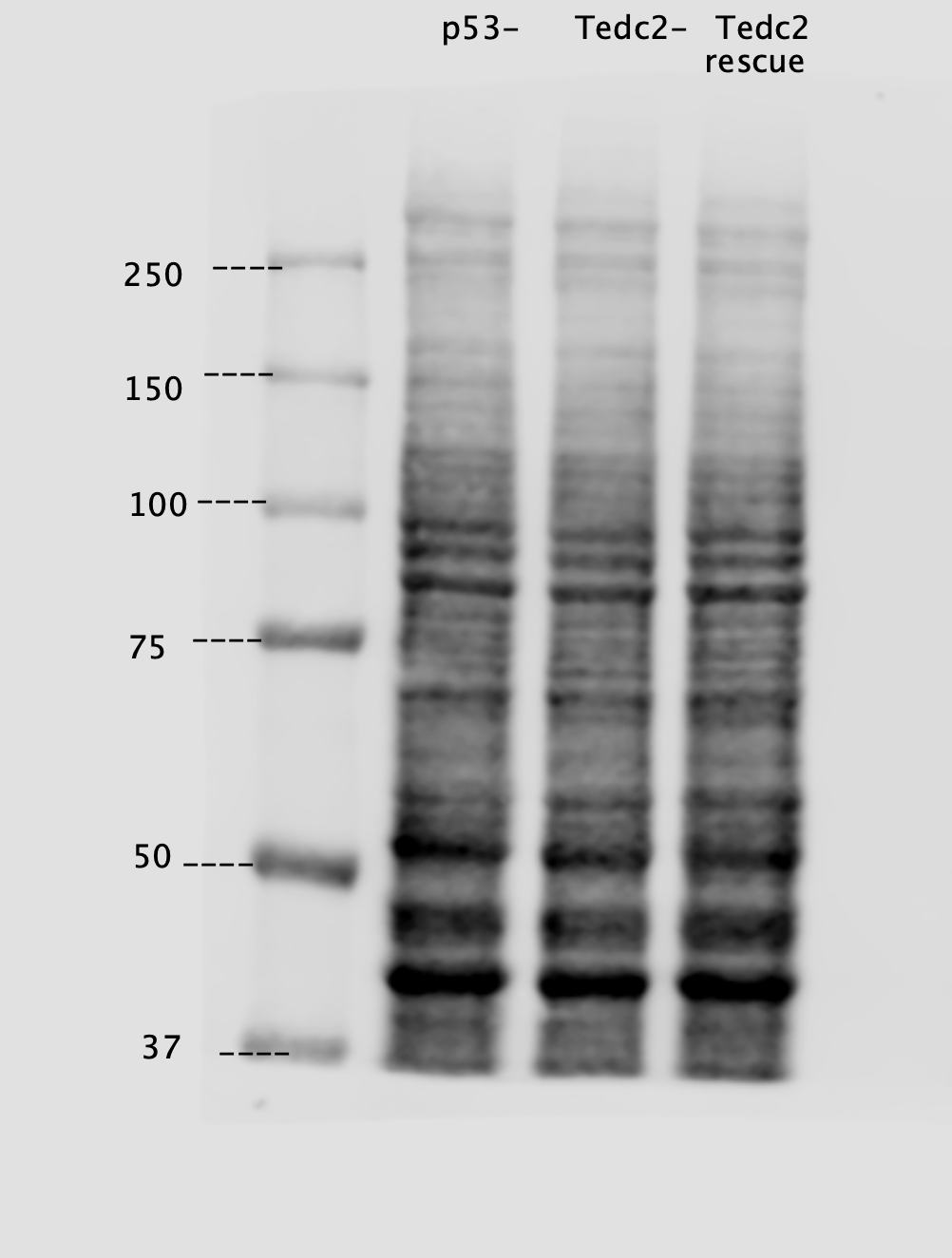

Supplement: Figure 1—figure supplement 1—source data 2. [file elife-98704-fig1-figsupp1-data2.zip › Fig 1 - Figure supplement 1 - source data 2/20240730-JWA13-Tedc2-revert-labeled.tif]

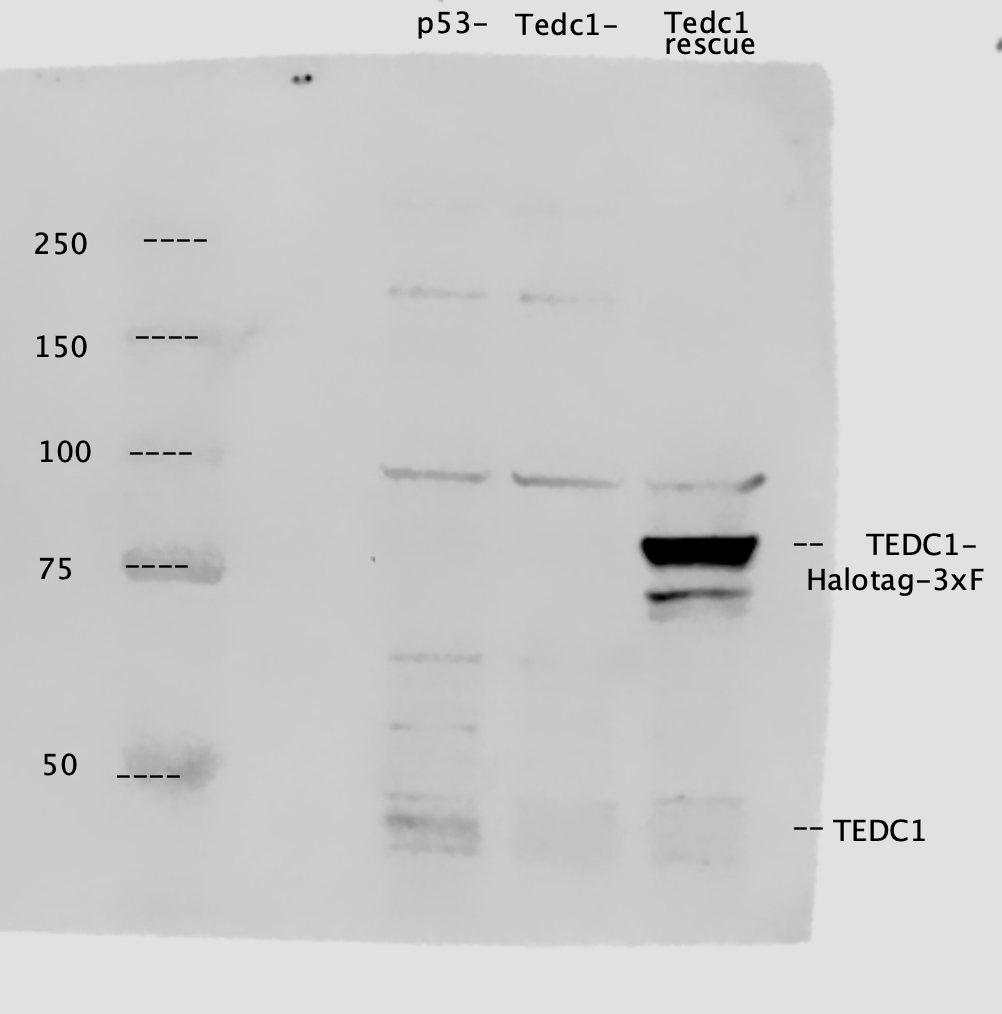

Supplement: Figure 1—figure supplement 1—source data 2. [file elife-98704-fig1-figsupp1-data2.zip › Fig 1 - Figure supplement 1 - source data 2/20240730-JWA13-Tedc1blot-labeled.tif]

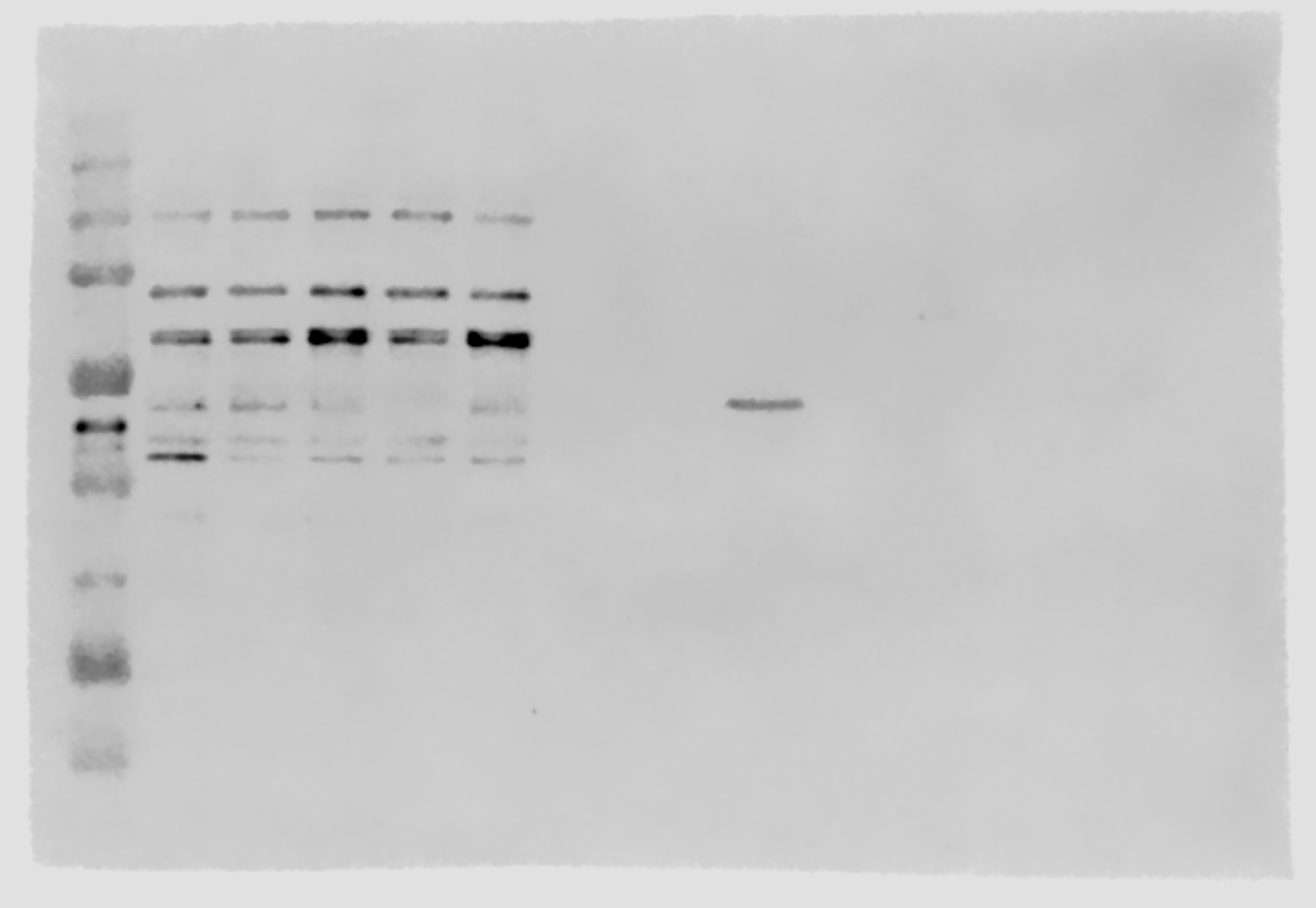

Supplement: Figure 3—source data 1. [file elife-98704-fig3-data1.zip › Fig 3 - source data 1/20231201_Fig3D_TUBE1blot_unlabeled.tif]

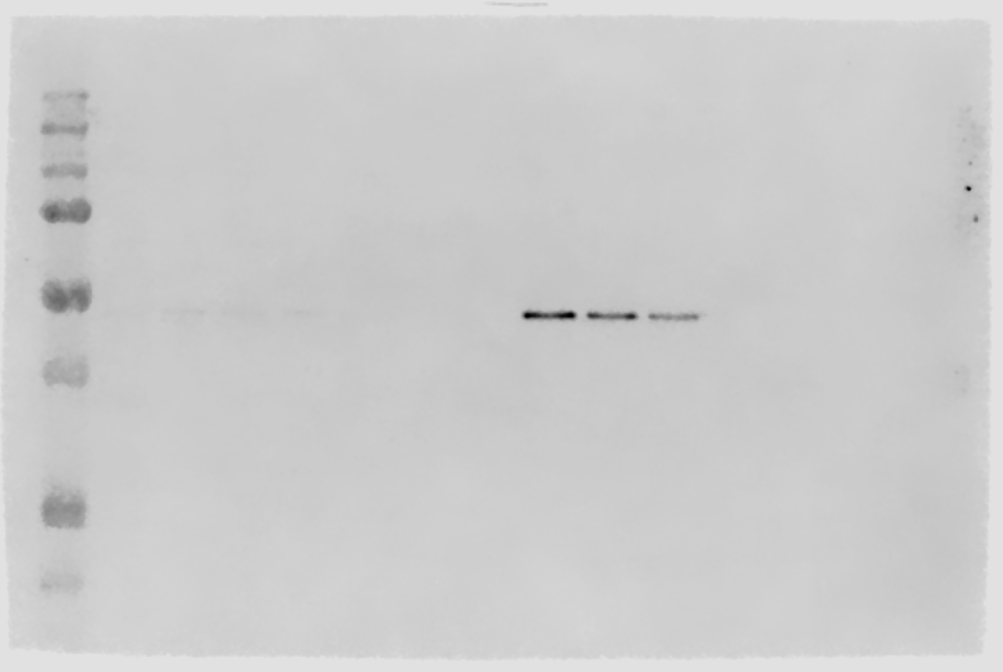

Supplement: Figure 3—source data 1. [file elife-98704-fig3-data1.zip › Fig 3 - source data 1/20231201_Fig3D_TEDC1blot_unlabeled.tif]

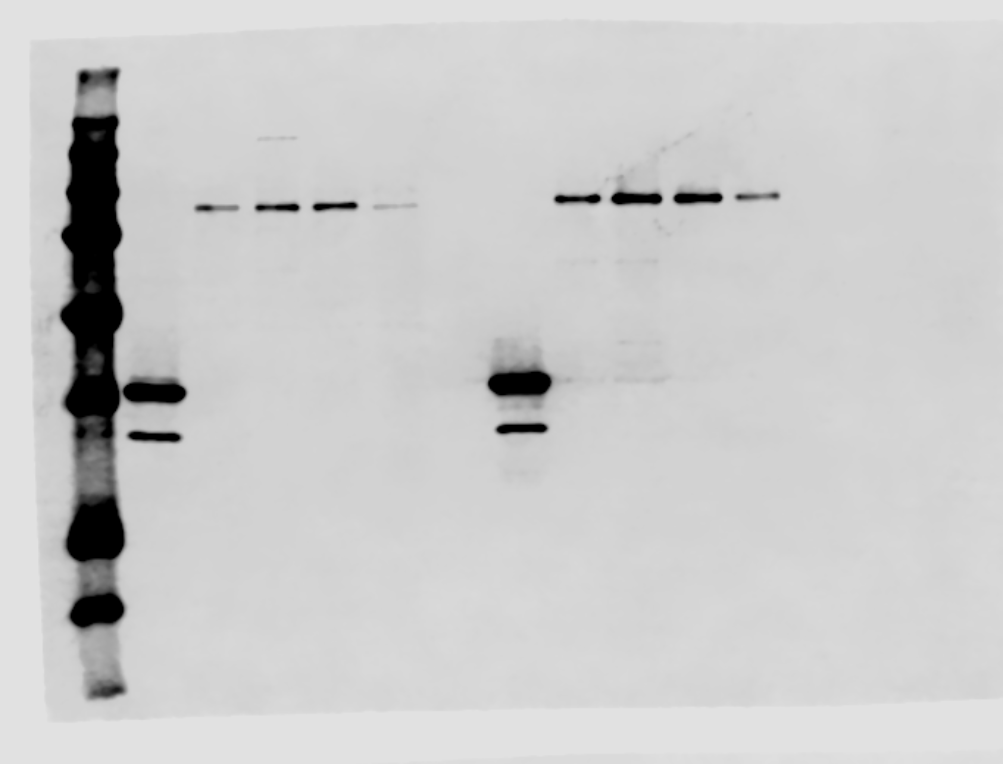

Supplement: Figure 3—source data 1. [file elife-98704-fig3-data1.zip › Fig 3 - source data 1/20231025_Fig3C_Flagblot_unlabeled.tif]

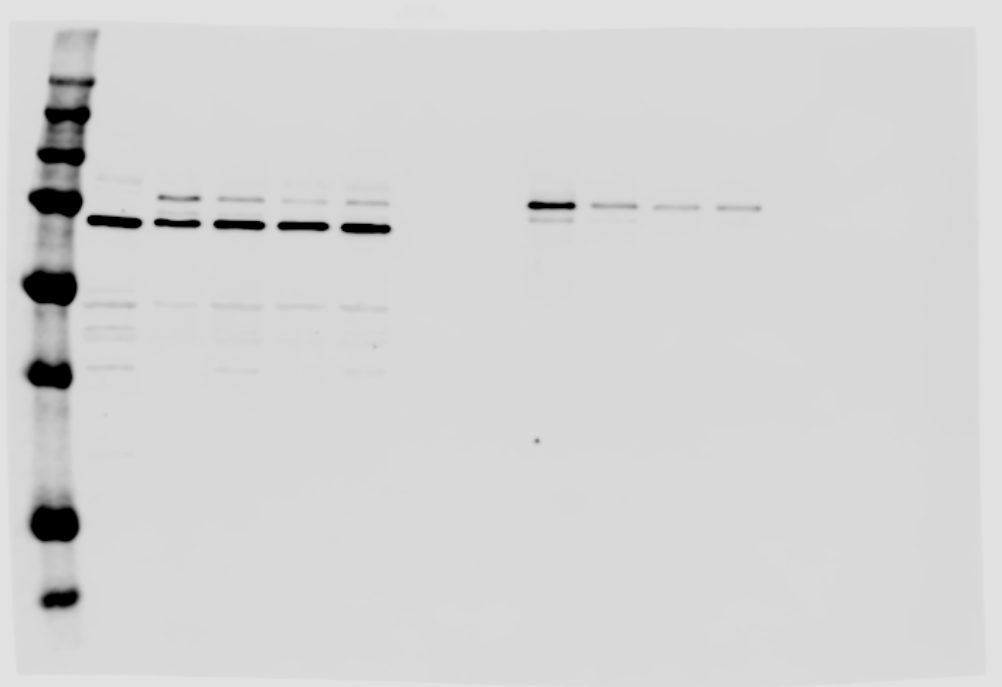

Supplement: Figure 3—source data 1. [file elife-98704-fig3-data1.zip › Fig 3 - source data 1/20231201_Fig3D_TEDC2blot_unlabeled.tif]

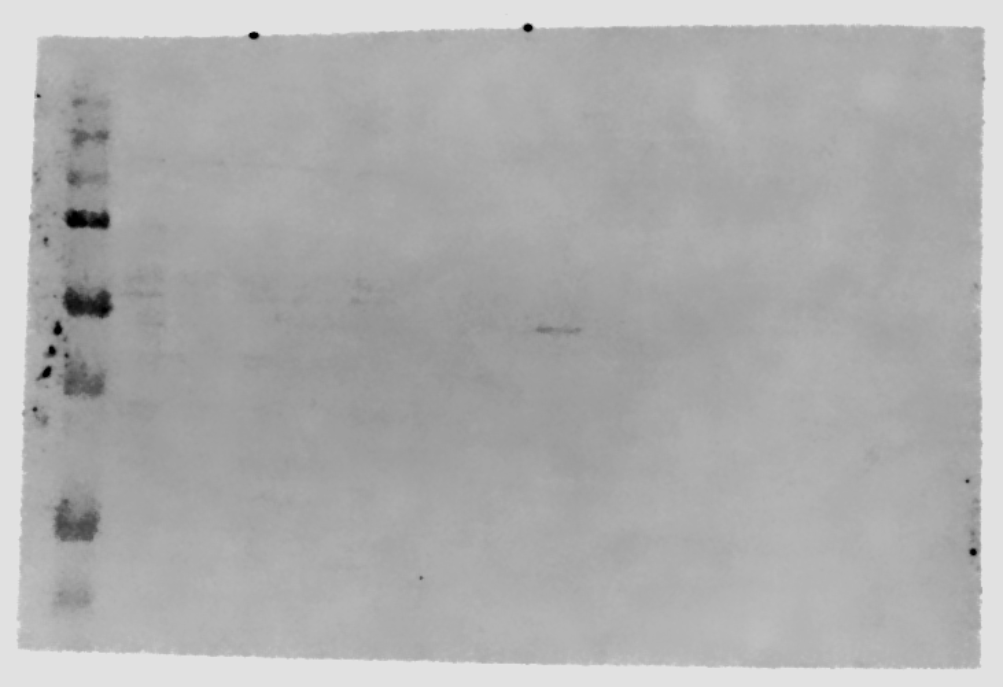

Supplement: Figure 3—source data 1. [file elife-98704-fig3-data1.zip › Fig 3 - source data 1/20231201_Fig3D_TUBD1lot_unlabeled.tif]

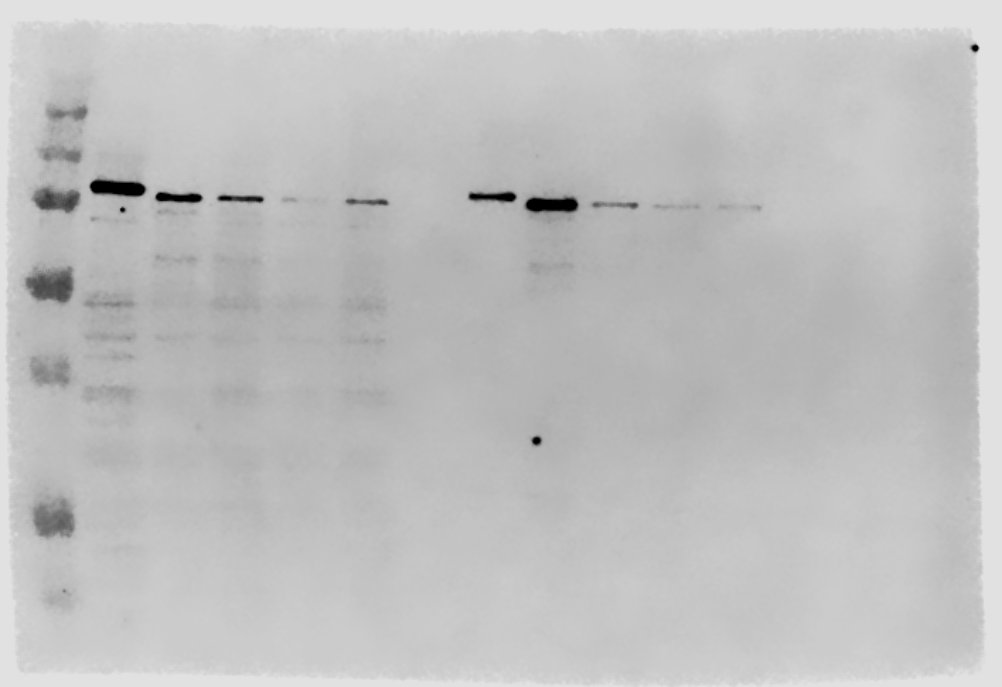

Supplement: Figure 3—source data 1. [file elife-98704-fig3-data1.zip › Fig 3 - source data 1/20231201_Fig3D_V5blot_unlabeled.tif]

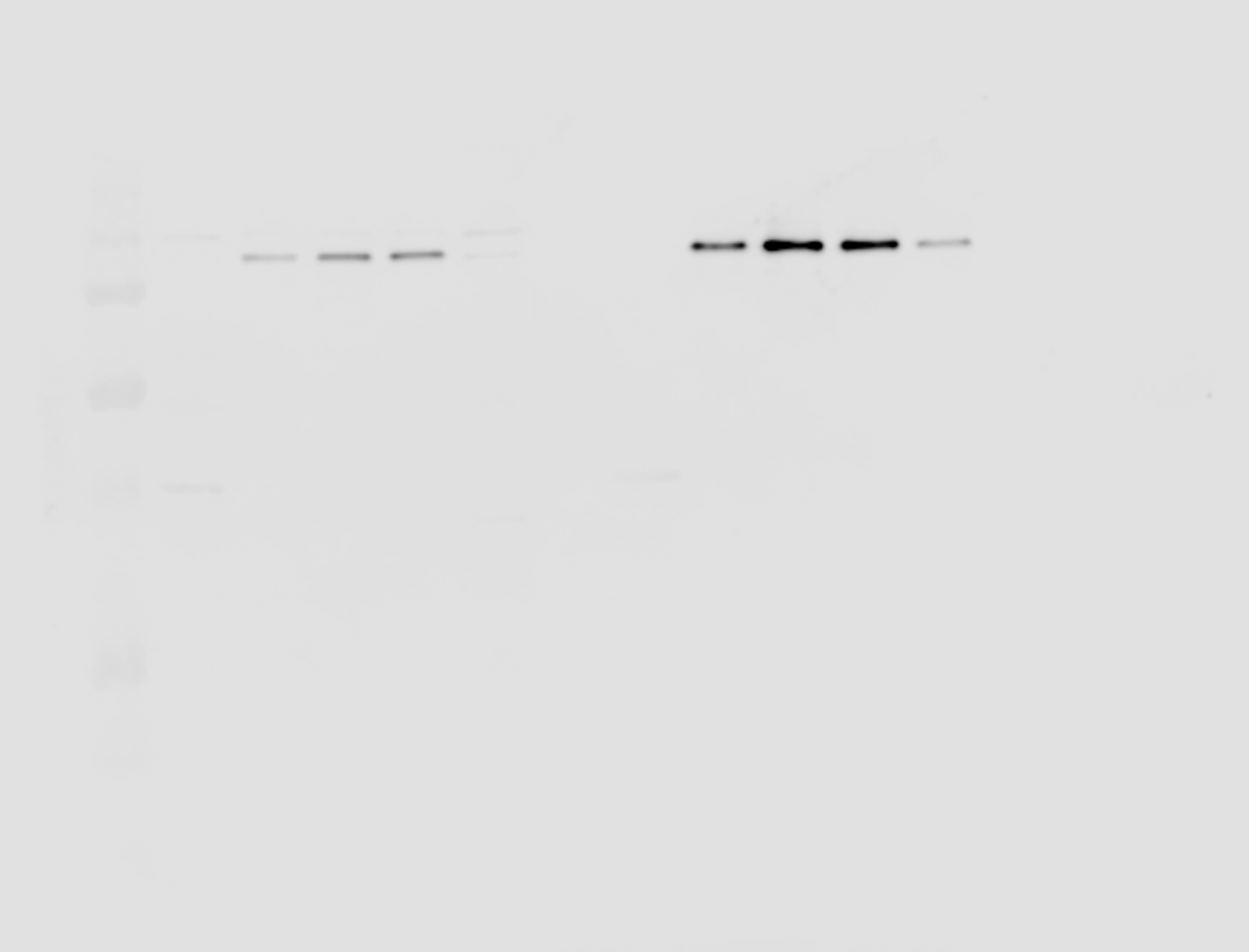

Supplement: Figure 3—source data 1. [file elife-98704-fig3-data1.zip › Fig 3 - source data 1/20231025_Fig3C_TEDC1blot_unlabeled.tif]

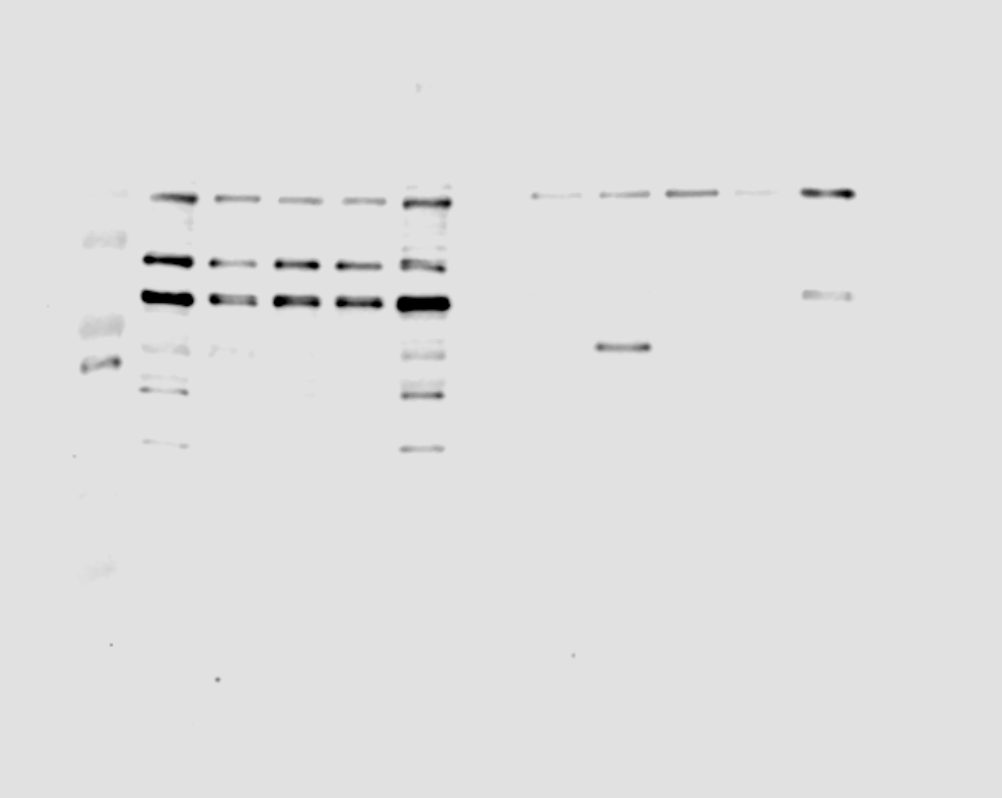

Supplement: Figure 3—source data 1. [file elife-98704-fig3-data1.zip › Fig 3 - source data 1/20231025_Fig3C_TUBE1blot_unlabeled.tif]

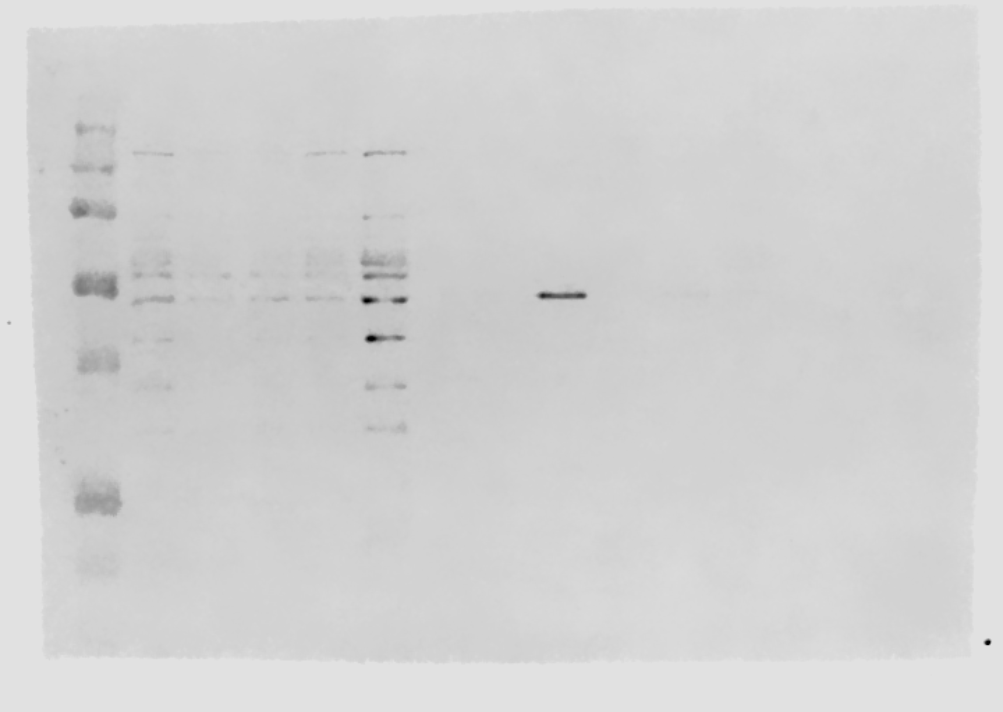

Supplement: Figure 3—source data 1. [file elife-98704-fig3-data1.zip › Fig 3 - source data 1/20231025_Fig3C_TUBD11blot_unlabeled.tif]

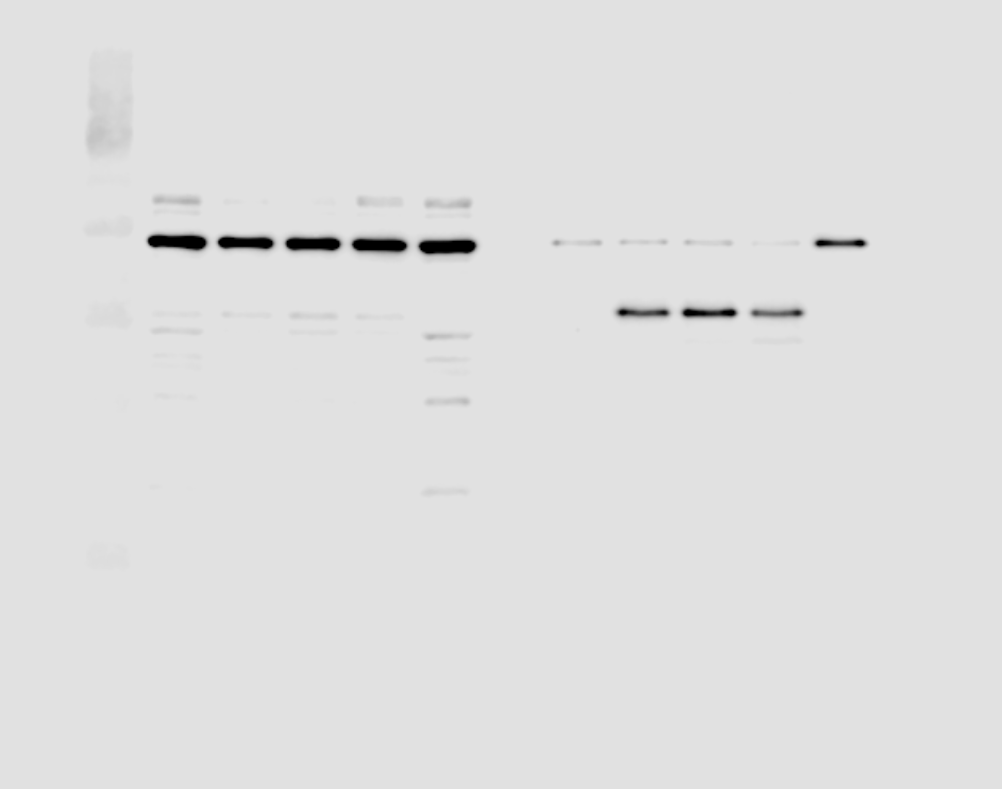

Supplement: Figure 3—source data 1. [file elife-98704-fig3-data1.zip › Fig 3 - source data 1/20231025_Fig3C_Tedc2blot_unlabeled.tif]

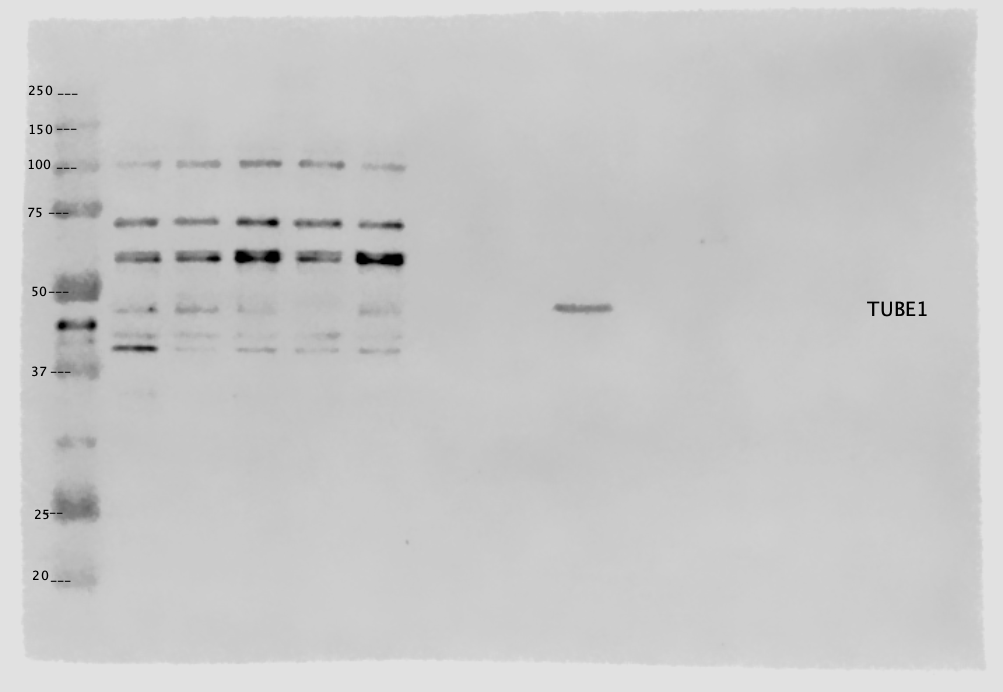

Supplement: Figure 3—source data 2. [file elife-98704-fig3-data2.zip › Fig 3 - source data 2/20231201_Fig3D_TUBE1blot_labeled.tif]

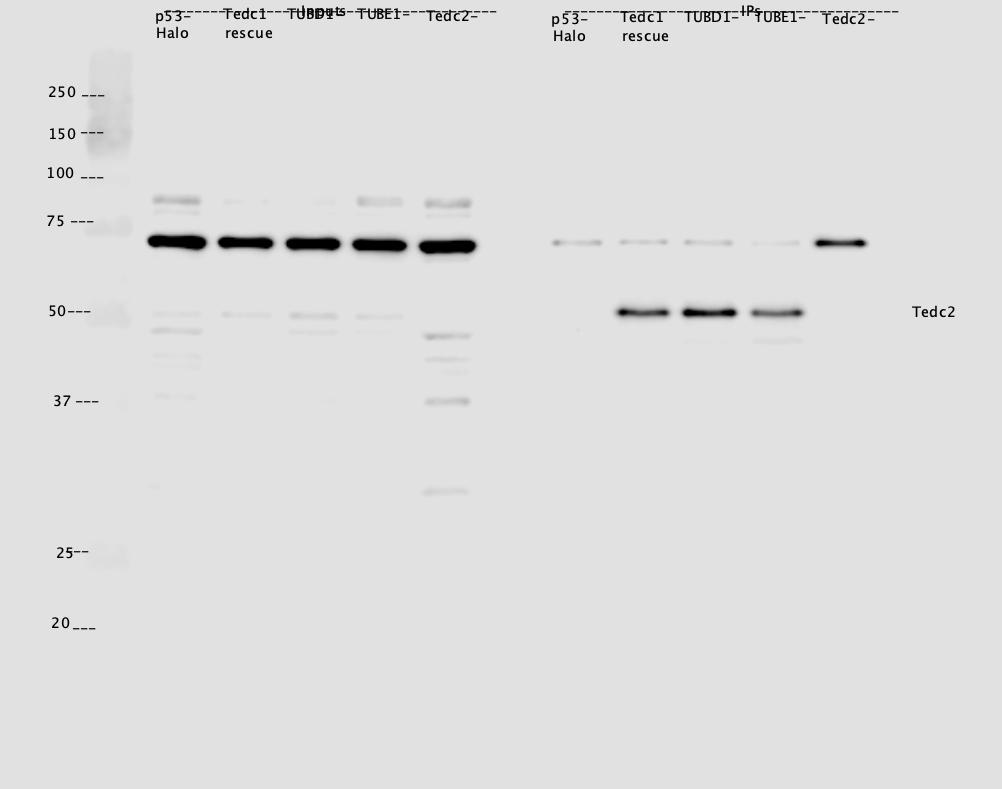

Supplement: Figure 3—source data 2. [file elife-98704-fig3-data2.zip › Fig 3 - source data 2/20231025_Fig3C_Tedc2blot_labeled.tif]

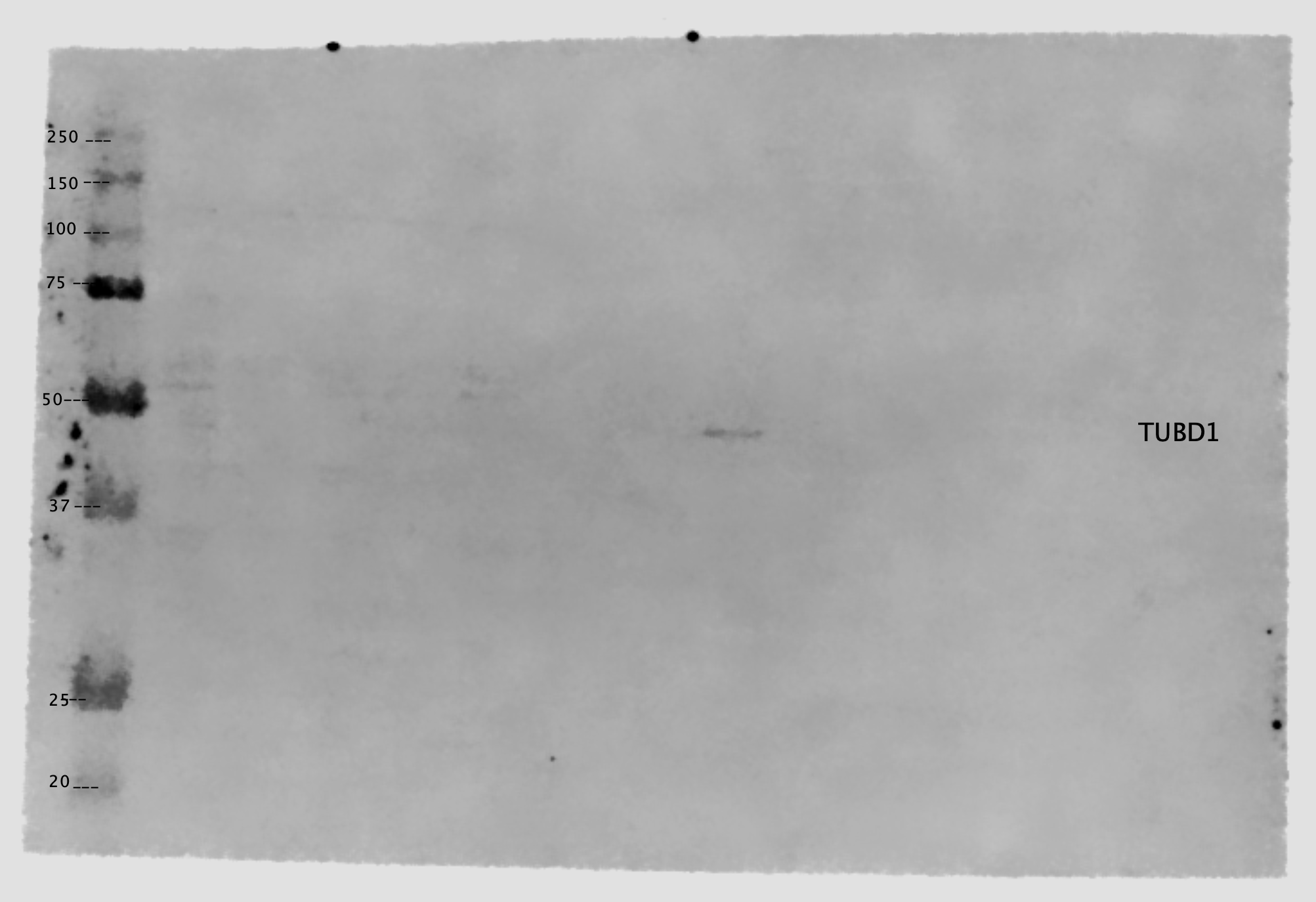

Supplement: Figure 3—source data 2. [file elife-98704-fig3-data2.zip › Fig 3 - source data 2/20231201_Fig3D_TUBD1lot_labeled.tif]

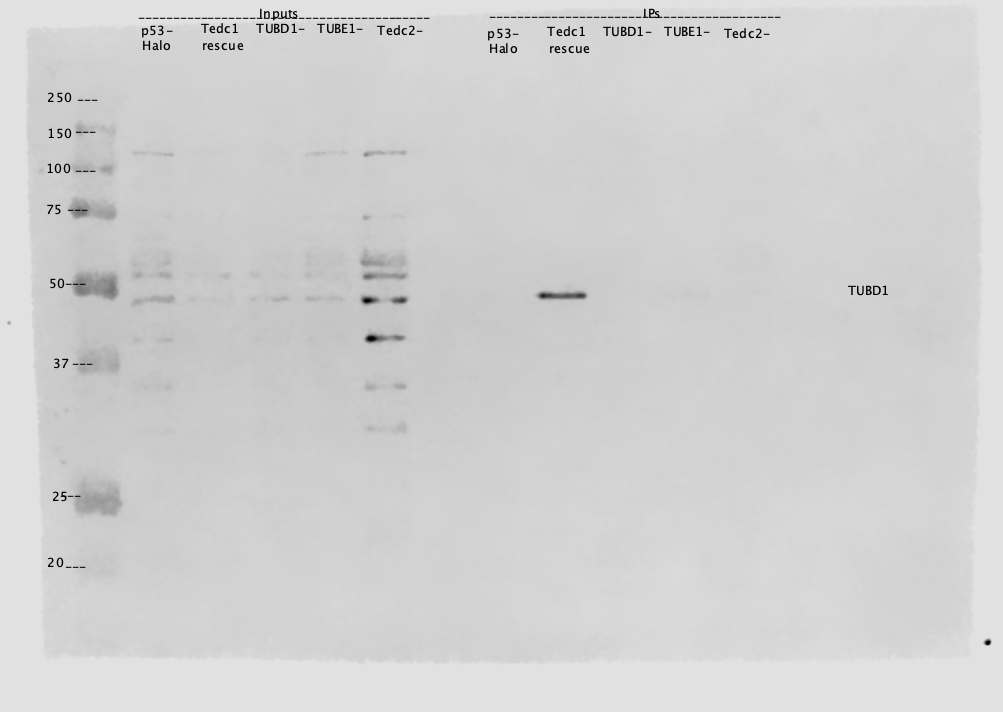

Supplement: Figure 3—source data 2. [file elife-98704-fig3-data2.zip › Fig 3 - source data 2/20231025_Fig3C_TUBD11blot_labeled.tif]

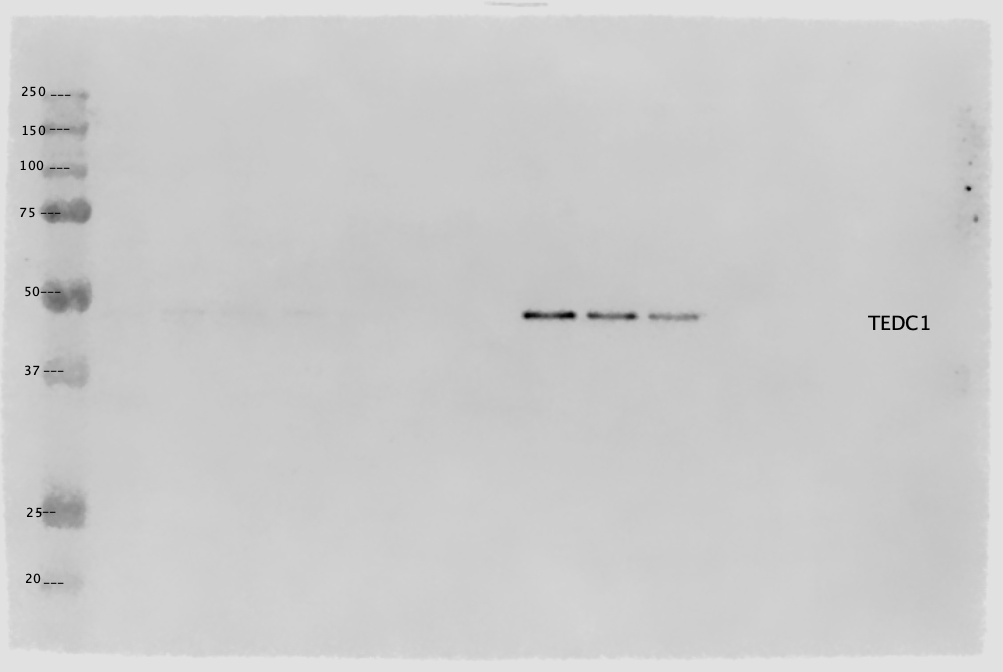

Supplement: Figure 3—source data 2. [file elife-98704-fig3-data2.zip › Fig 3 - source data 2/20231201_Fig3D_TEDC1blot_labeled.tif]

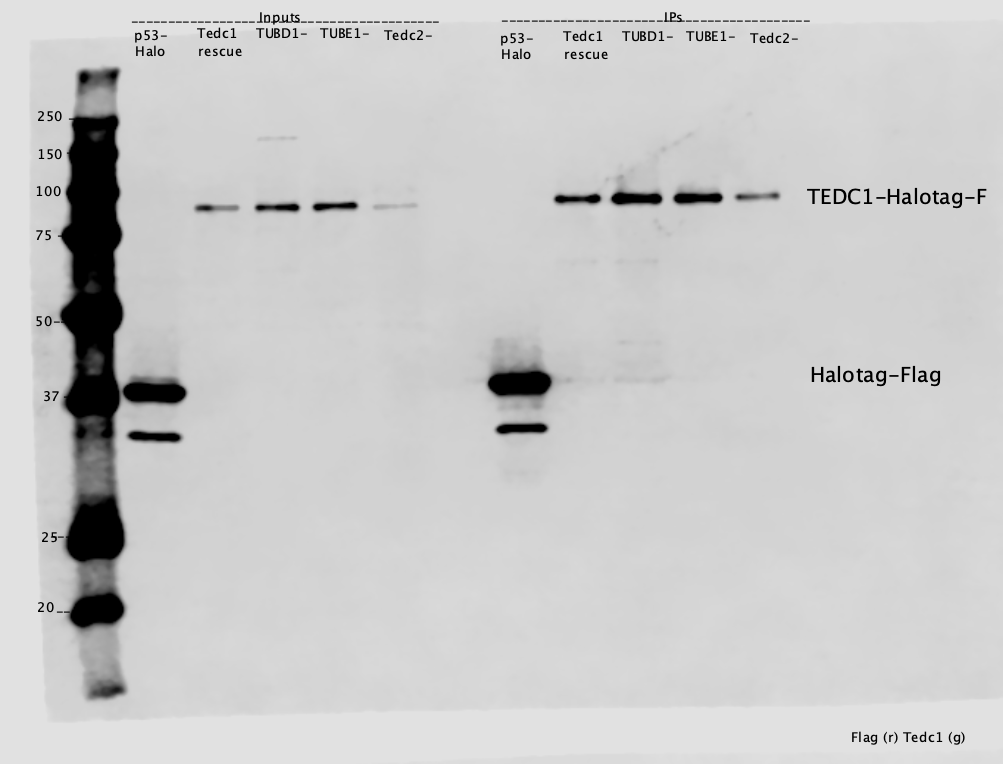

Supplement: Figure 3—source data 2. [file elife-98704-fig3-data2.zip › Fig 3 - source data 2/20231025_Fig3C_Flagblot_labeled.tif]

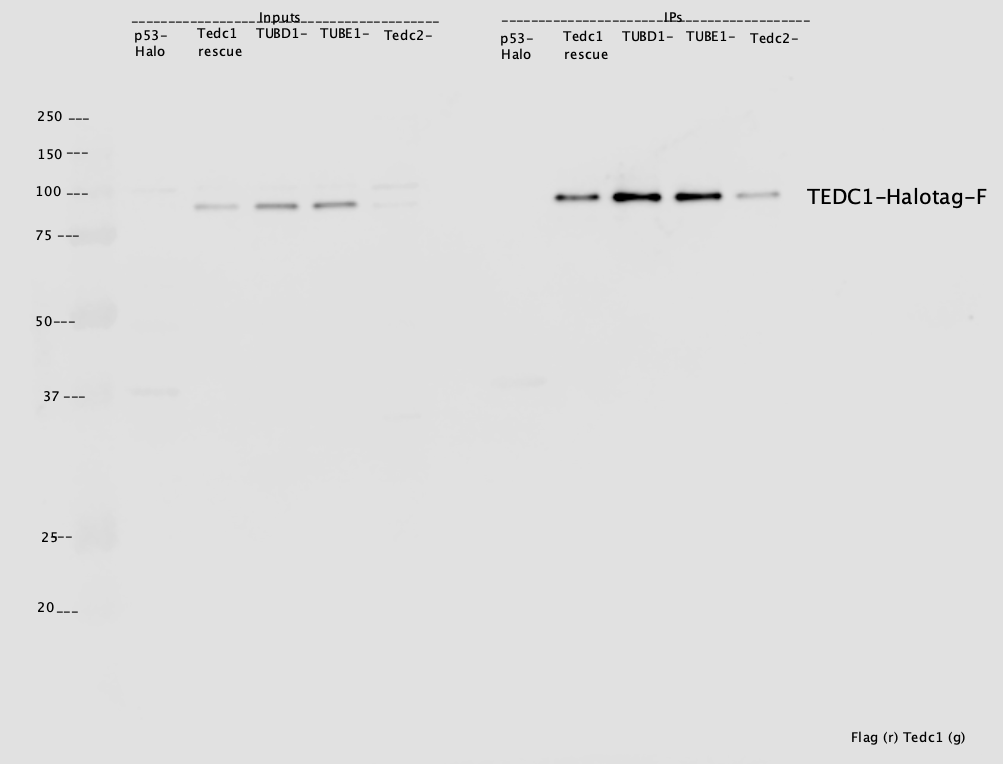

Supplement: Figure 3—source data 2. [file elife-98704-fig3-data2.zip › Fig 3 - source data 2/20231025_Fig3C_TEDC1blot_labeled.tif]

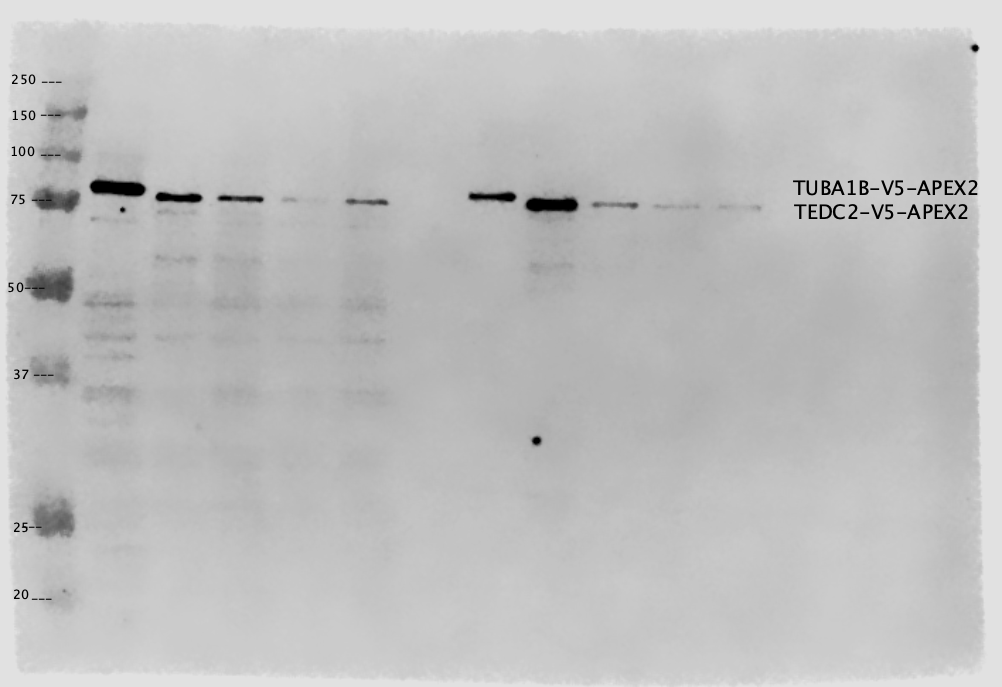

Supplement: Figure 3—source data 2. [file elife-98704-fig3-data2.zip › Fig 3 - source data 2/20231201_Fig3D_V5blot_labeled.tif]

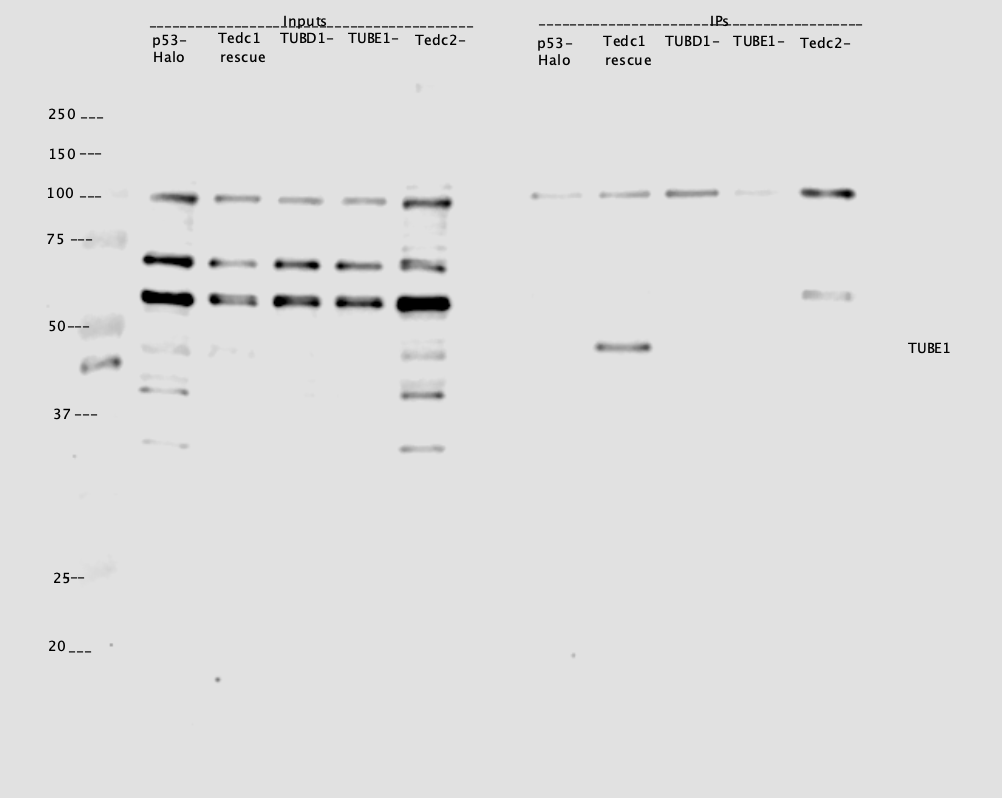

Supplement: Figure 3—source data 2. [file elife-98704-fig3-data2.zip › Fig 3 - source data 2/20231025_Fig3C_TUBE1blot_labeled.tif]

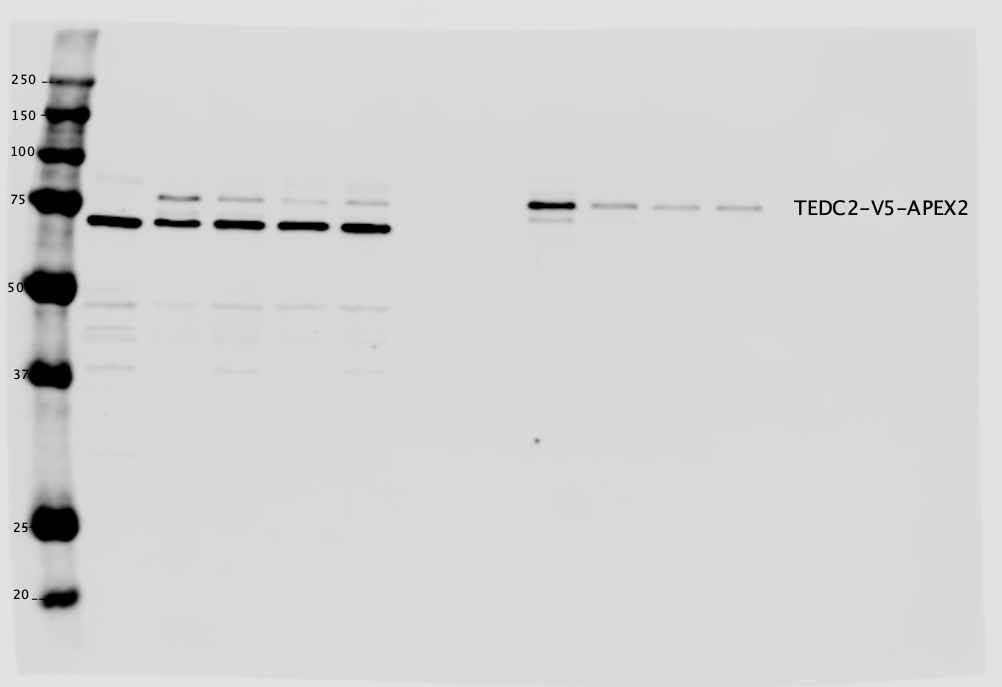

Supplement: Figure 3—source data 2. [file elife-98704-fig3-data2.zip › Fig 3 - source data 2/20231201_Fig3D_TEDC2blot_labeled.tif]

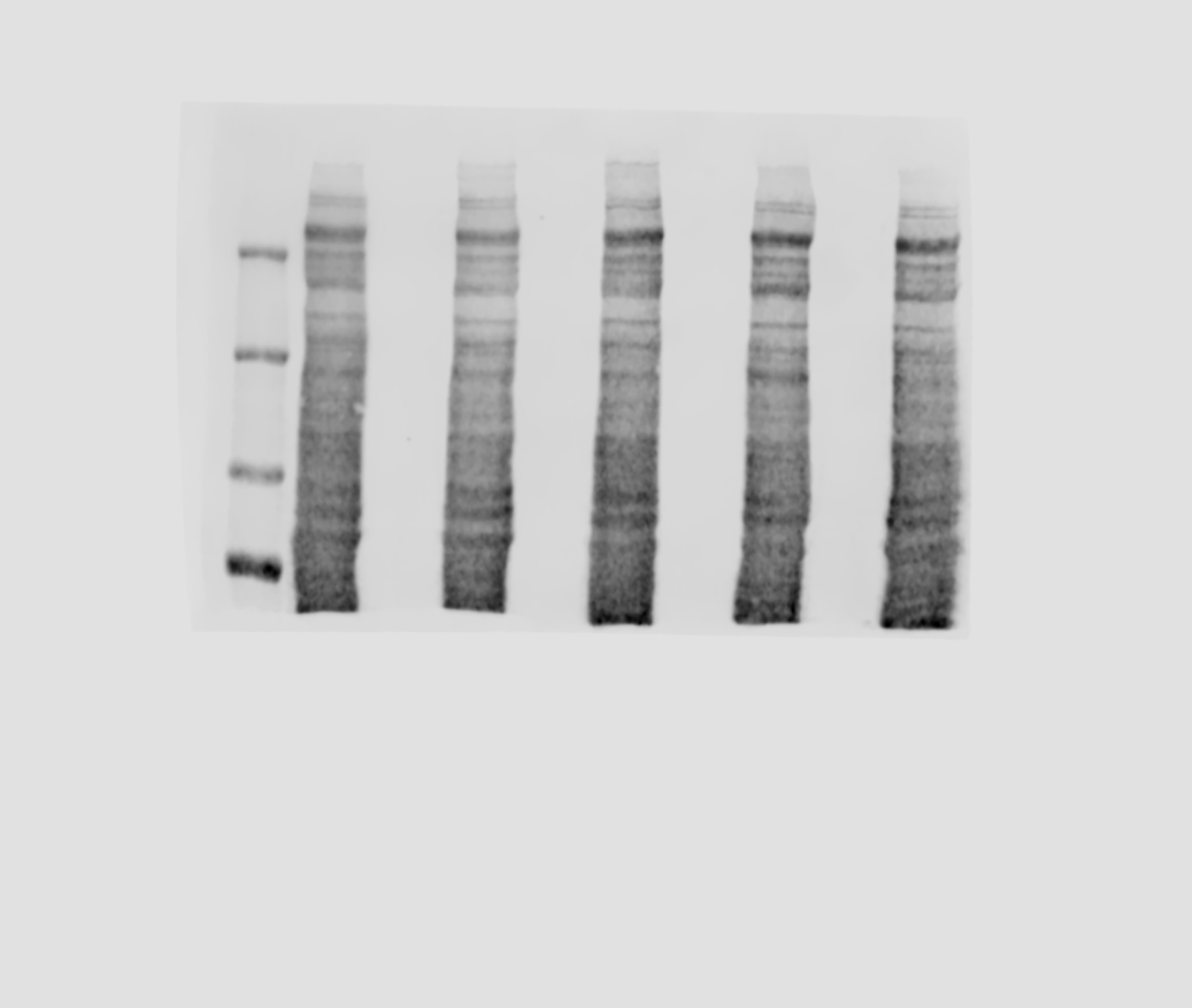

Supplement: Figure 4—figure supplement 2—source data 1. [file elife-98704-fig4-figsupp2-data1.zip › Fig 4 - Figure supplement 2 - source data 1/STIL-Revert.tif]

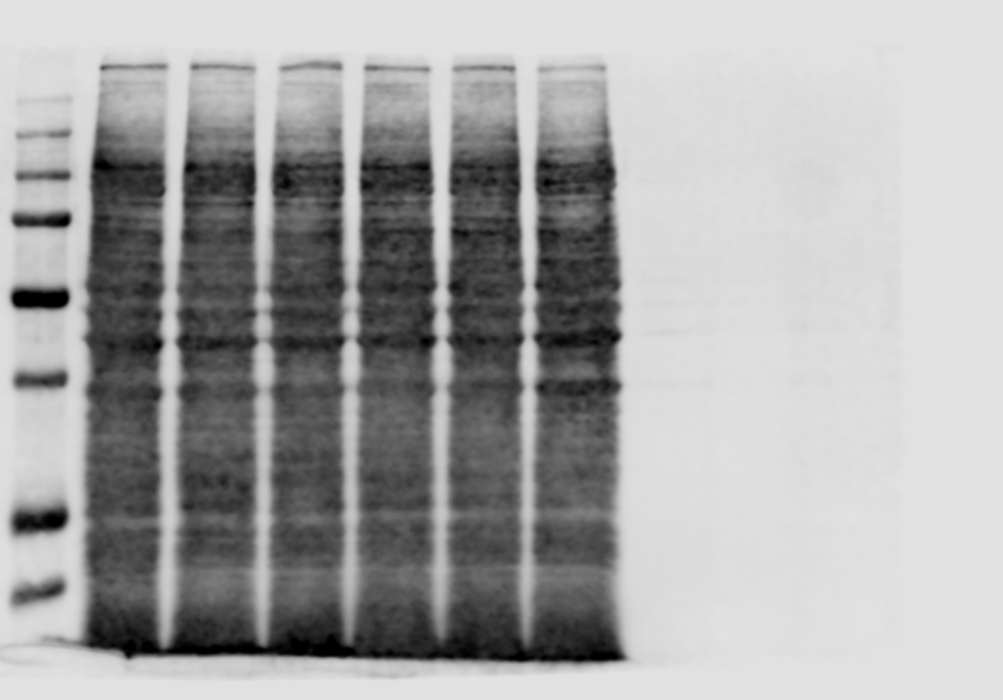

Supplement: Figure 4—figure supplement 2—source data 1. [file elife-98704-fig4-figsupp2-data1.zip › Fig 4 - Figure supplement 2 - source data 1/SASS6-Revert.tif]

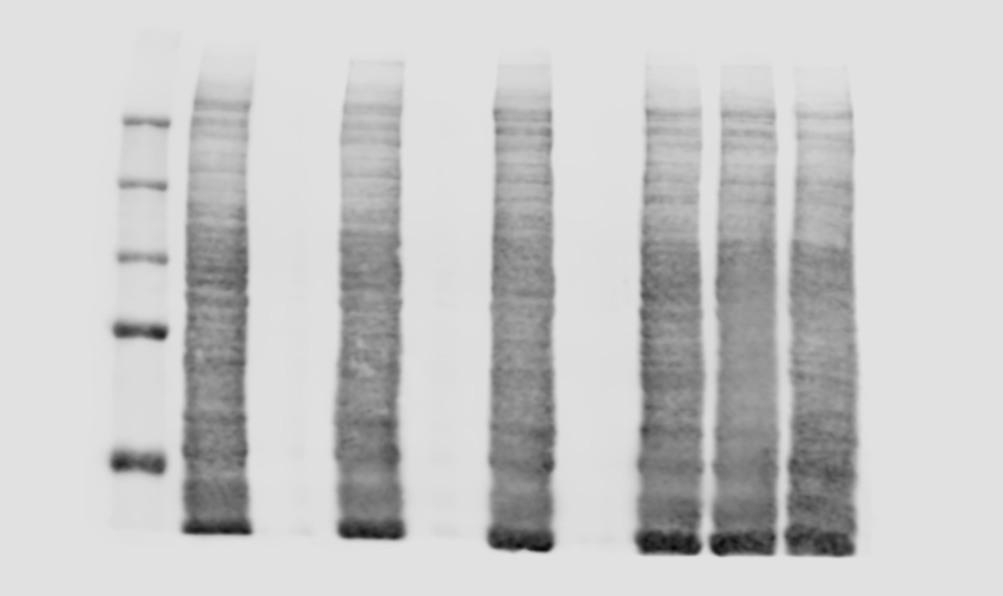

Supplement: Figure 4—figure supplement 2—source data 1. [file elife-98704-fig4-figsupp2-data1.zip › Fig 4 - Figure supplement 2 - source data 1/CPAP-Revert.tif]

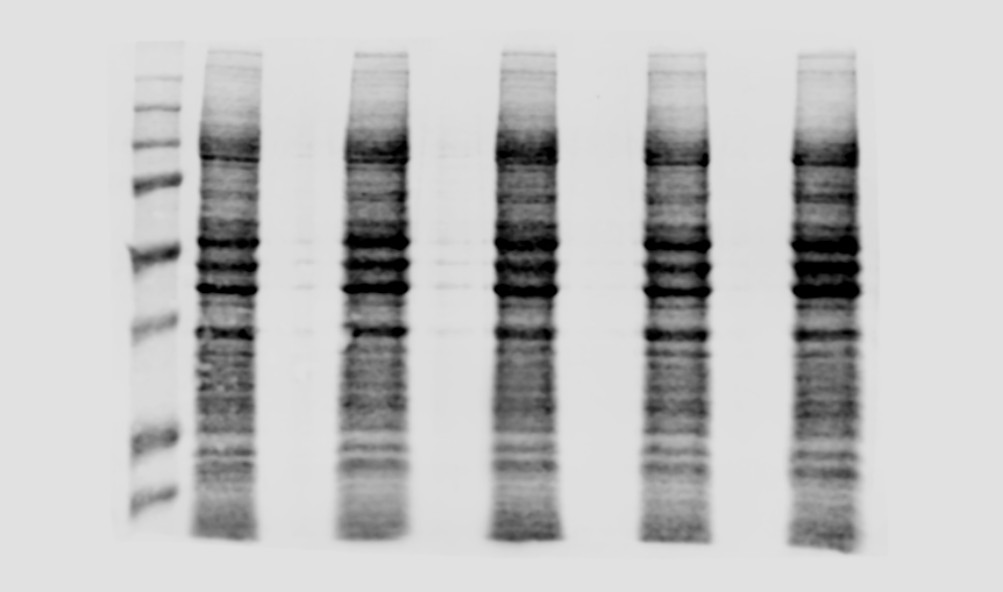

Supplement: Figure 4—figure supplement 2—source data 1. [file elife-98704-fig4-figsupp2-data1.zip › Fig 4 - Figure supplement 2 - source data 1/POC5-revert.tif]

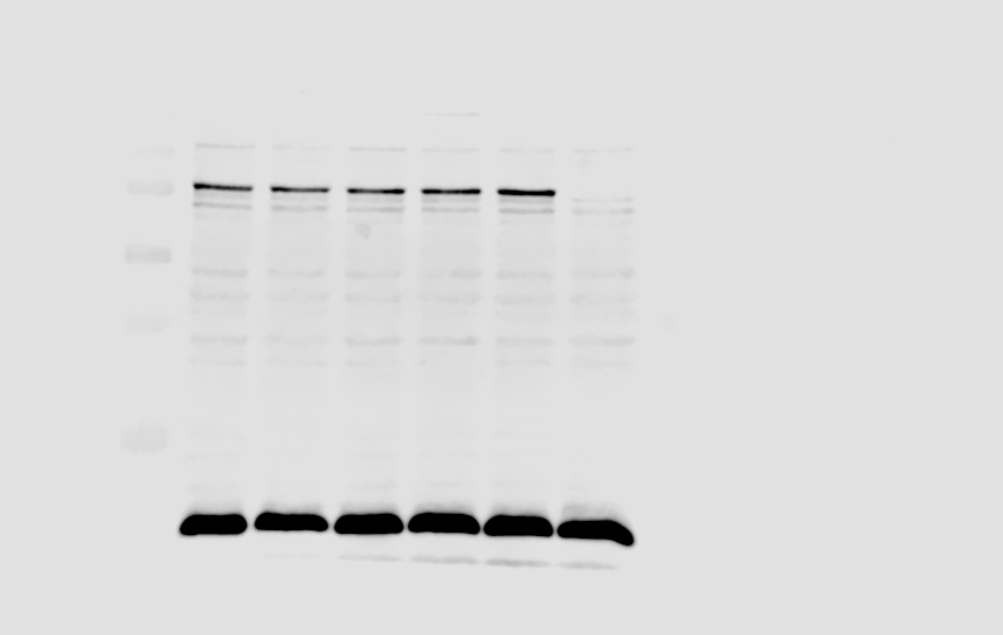

Supplement: Figure 4—figure supplement 2—source data 1. [file elife-98704-fig4-figsupp2-data1.zip › Fig 4 - Figure supplement 2 - source data 1/SASS6.tif]

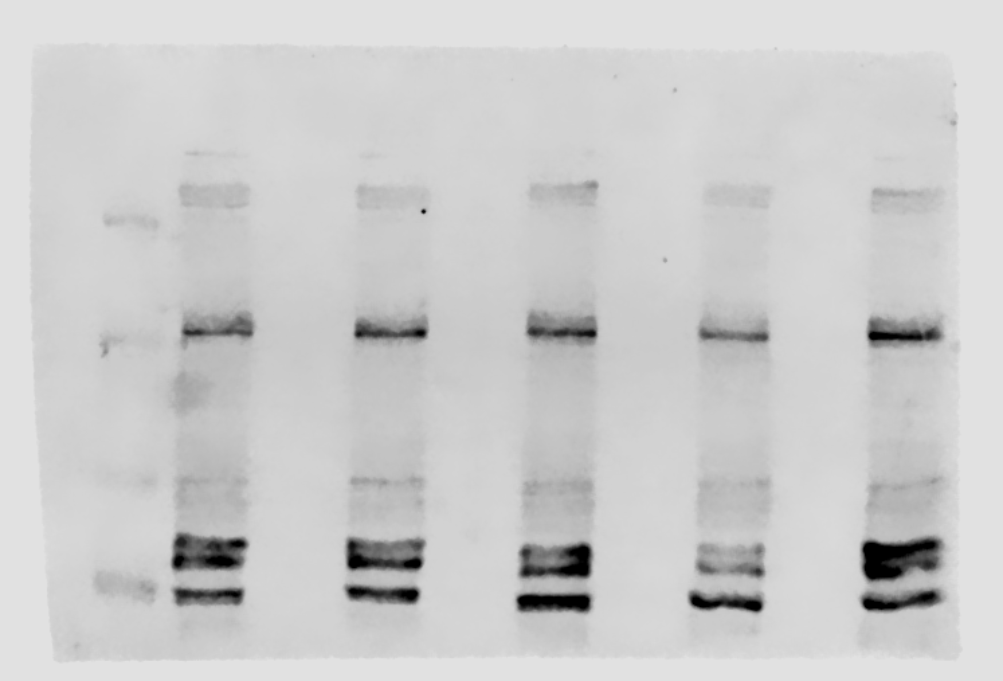

Supplement: Figure 4—figure supplement 2—source data 1. [file elife-98704-fig4-figsupp2-data1.zip › Fig 4 - Figure supplement 2 - source data 1/STIL.tif]

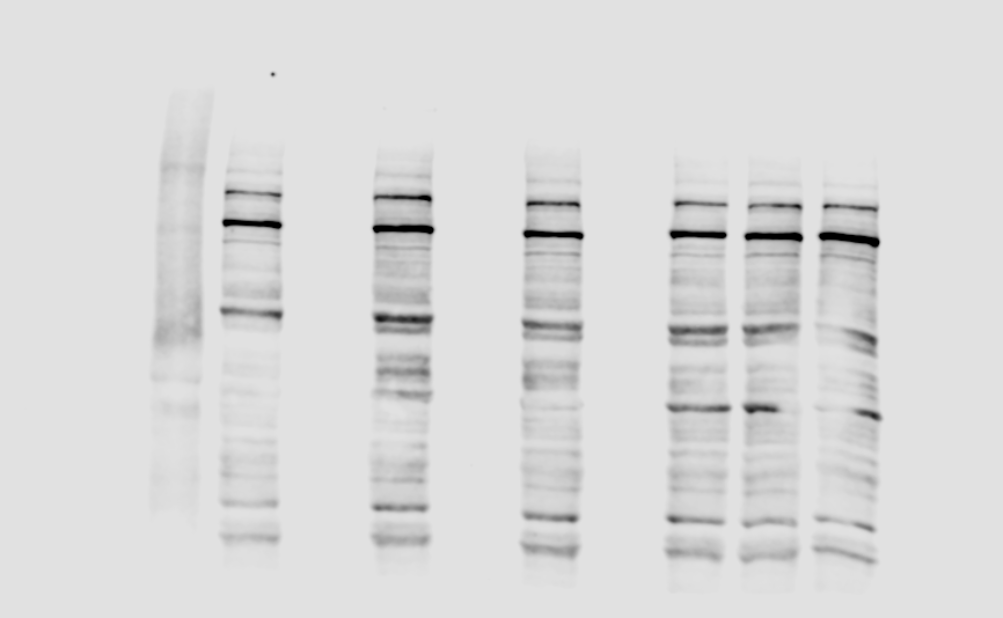

Supplement: Figure 4—figure supplement 2—source data 1. [file elife-98704-fig4-figsupp2-data1.zip › Fig 4 - Figure supplement 2 - source data 1/CPAP.tif]

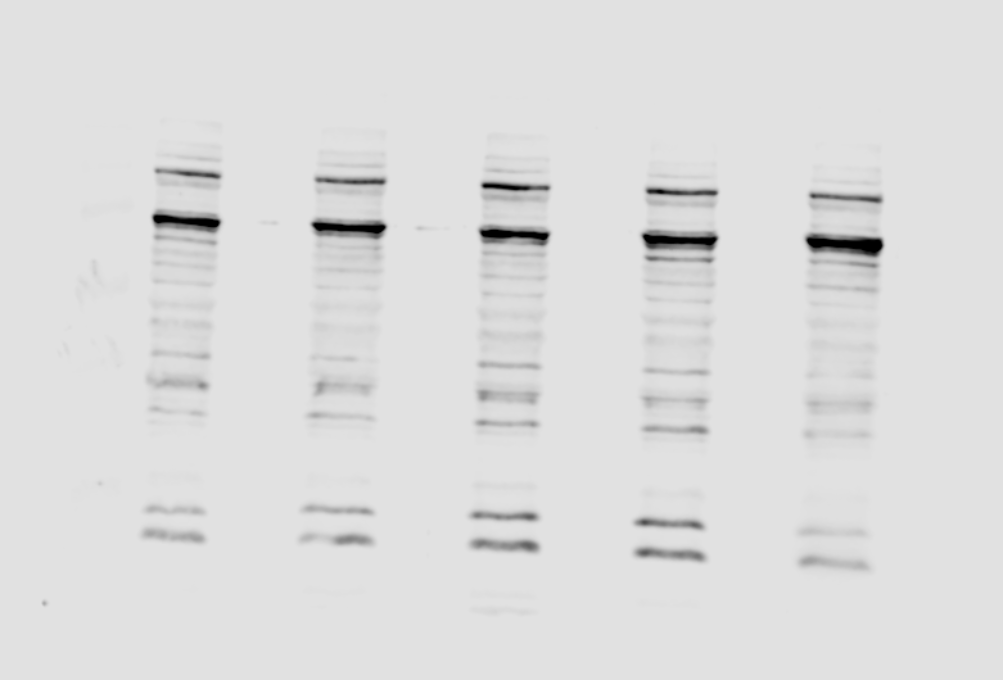

Supplement: Figure 4—figure supplement 2—source data 1. [file elife-98704-fig4-figsupp2-data1.zip › Fig 4 - Figure supplement 2 - source data 1/POC5.tif]

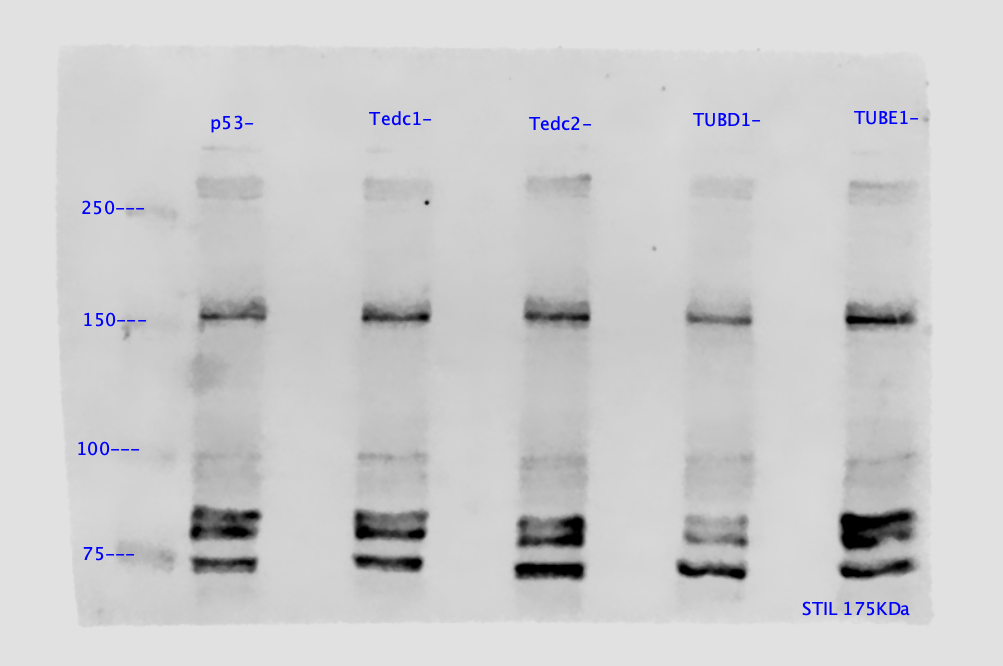

Supplement: Figure 4—figure supplement 2—source data 2. [file elife-98704-fig4-figsupp2-data2.zip › Fig 4 - Figure supplement 2 - source data 2/LXB2-STIL.tif]

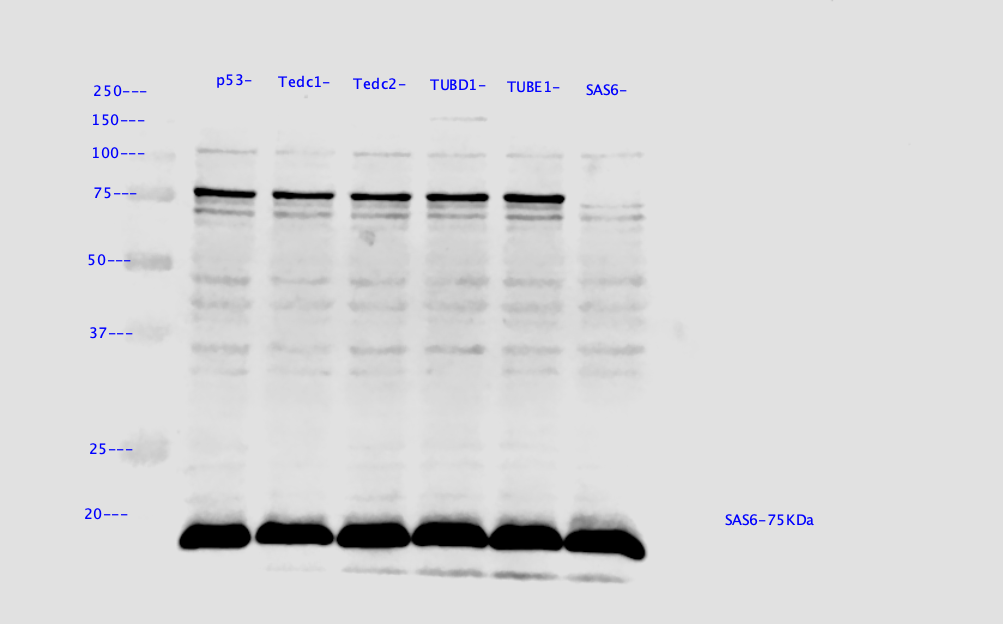

Supplement: Figure 4—figure supplement 2—source data 2. [file elife-98704-fig4-figsupp2-data2.zip › Fig 4 - Figure supplement 2 - source data 2/LXB1-SAS6.tif]

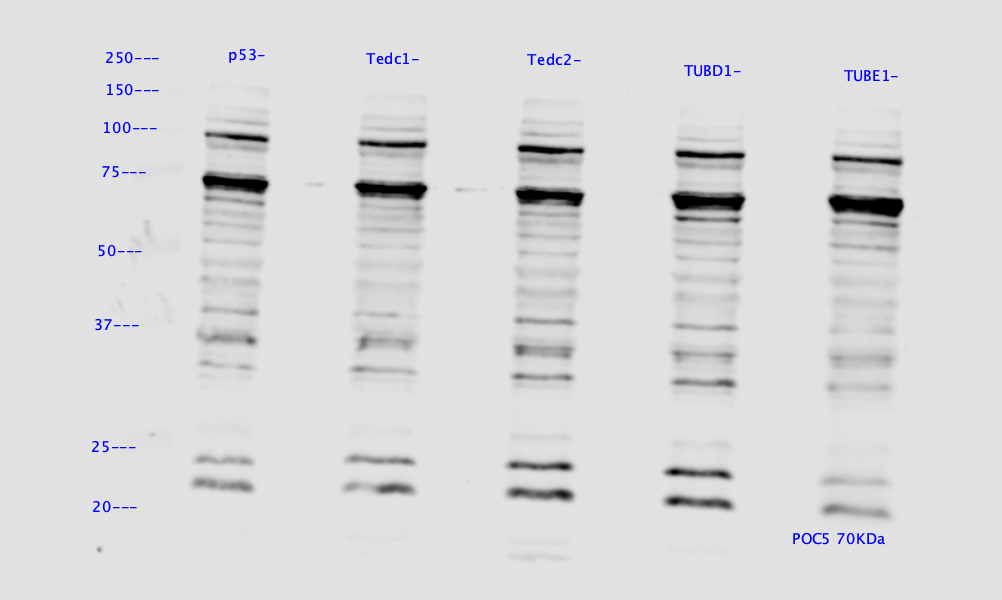

Supplement: Figure 4—figure supplement 2—source data 2. [file elife-98704-fig4-figsupp2-data2.zip › Fig 4 - Figure supplement 2 - source data 2/LXB5-POC5.tif]

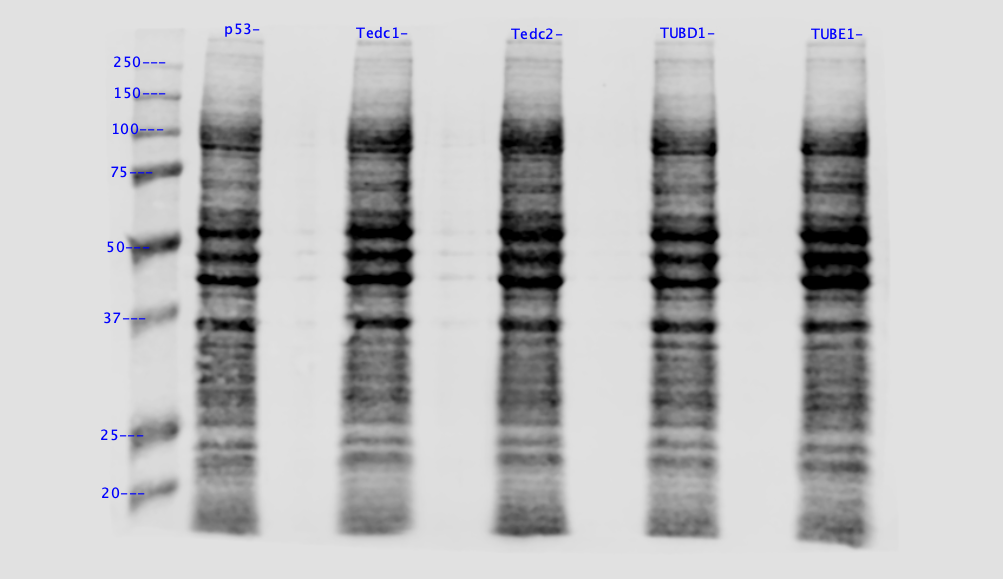

Supplement: Figure 4—figure supplement 2—source data 2. [file elife-98704-fig4-figsupp2-data2.zip › Fig 4 - Figure supplement 2 - source data 2/LXB5-POC5 revert.tif]

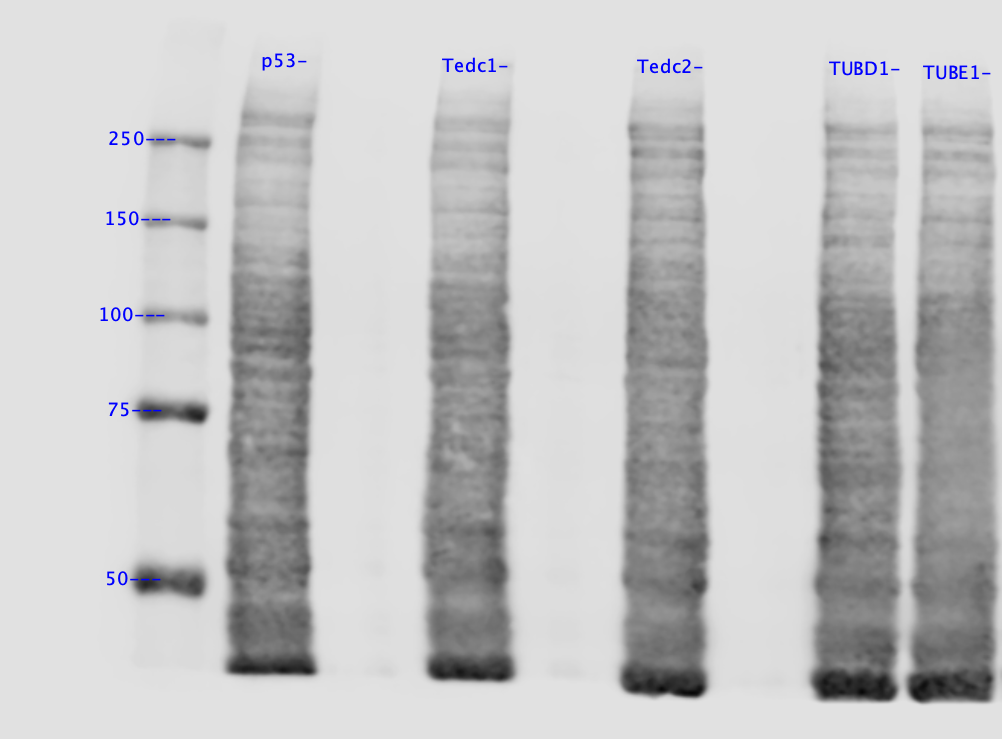

Supplement: Figure 4—figure supplement 2—source data 2. [file elife-98704-fig4-figsupp2-data2.zip › Fig 4 - Figure supplement 2 - source data 2/CPAP revert.tif]

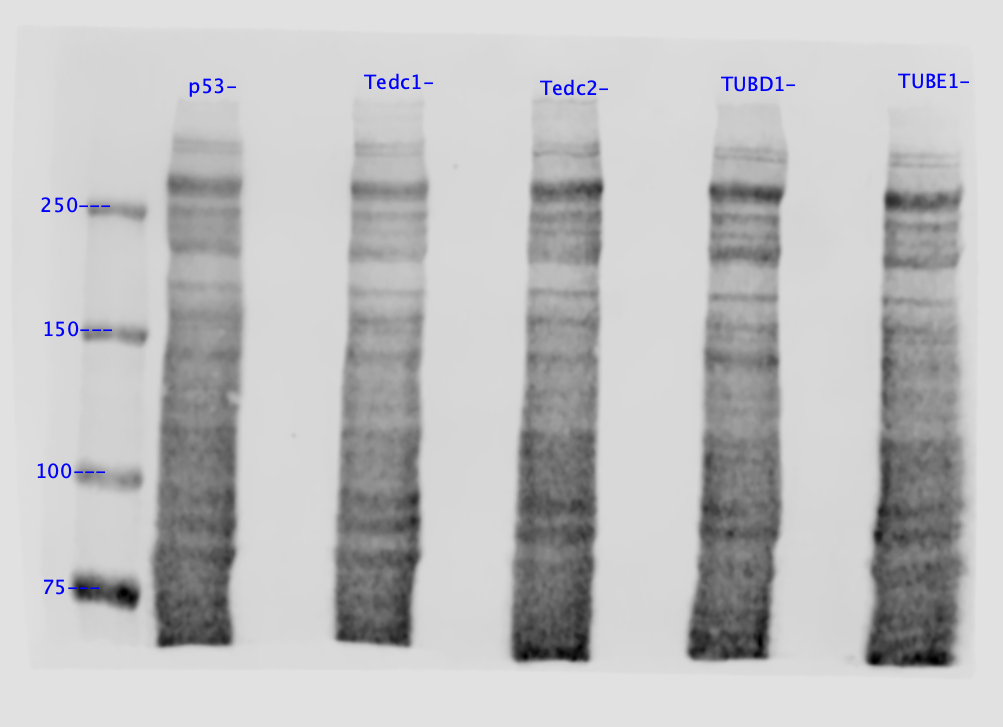

Supplement: Figure 4—figure supplement 2—source data 2. [file elife-98704-fig4-figsupp2-data2.zip › Fig 4 - Figure supplement 2 - source data 2/LXB2-STIL revert.tif]

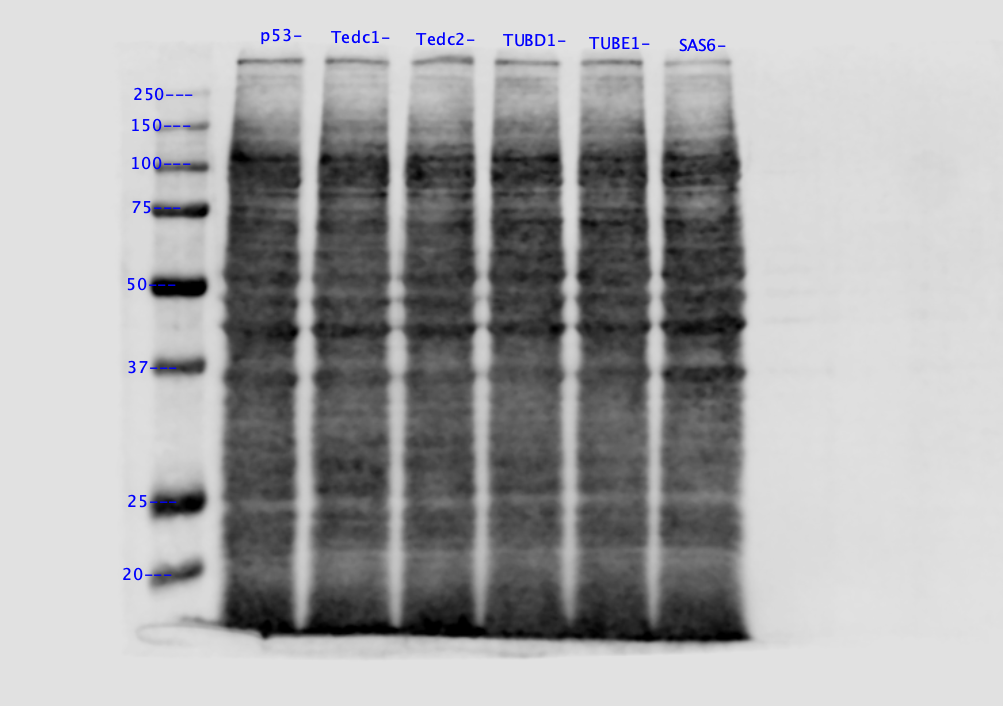

Supplement: Figure 4—figure supplement 2—source data 2. [file elife-98704-fig4-figsupp2-data2.zip › Fig 4 - Figure supplement 2 - source data 2/LXB1-SAS6 revert.tif]

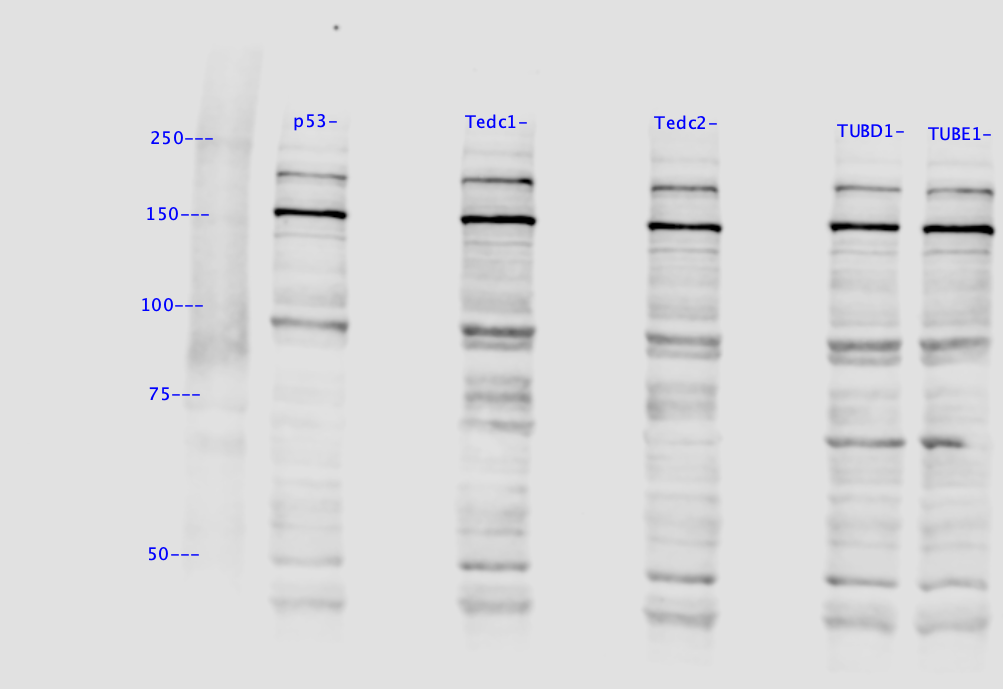

Supplement: Figure 4—figure supplement 2—source data 2. [file elife-98704-fig4-figsupp2-data2.zip › Fig 4 - Figure supplement 2 - source data 2/CPAP.tif]
